# Supplementary material for: Women with polycystic ovary syndrome exhibit impaired endometrial receptivity with excessive ERα and histone lactylation
Source: Nat Commun. 2026 Jan 21;17:1739. doi: 10.1038/s41467-026-68441-0 (PMC12913789; doi:10.1038/s41467-026-68441-0)
Supplement: Supplementary file 1 — Supplementary Information [file 41467_2026_68441_MOESM1_ESM.pdf]

Supplementary Information for

Women with polycystic ovary syndrome exhibit impaired endometrial receptivity with excessive ERα and histone lactylation

Hongying Shan*, Yue Wang*, Baoying Liao*, Kai-Lun Hu*, Xiunan Chen, Chenxi Xiao, Zi Yang, Fenting Liu, Tianliu Peng, Mingmei Lin, Feng Deng, Ping Zhou#, Yang Yu#, Rong Li#, Heng Pan#

* These authors contributed equally to this work.

# Corresponding authors. Ping Zhou, zhoup0520@163.com;

Yang Yu, yuyang5012@hotmail.com; Rong Li, roseli001@sina.com;

Heng Pan, hep2007@bjmu.edu.cn.

This PDF file includes:

Supplementary Figs. 1-8

Supplementary Tables 1-4

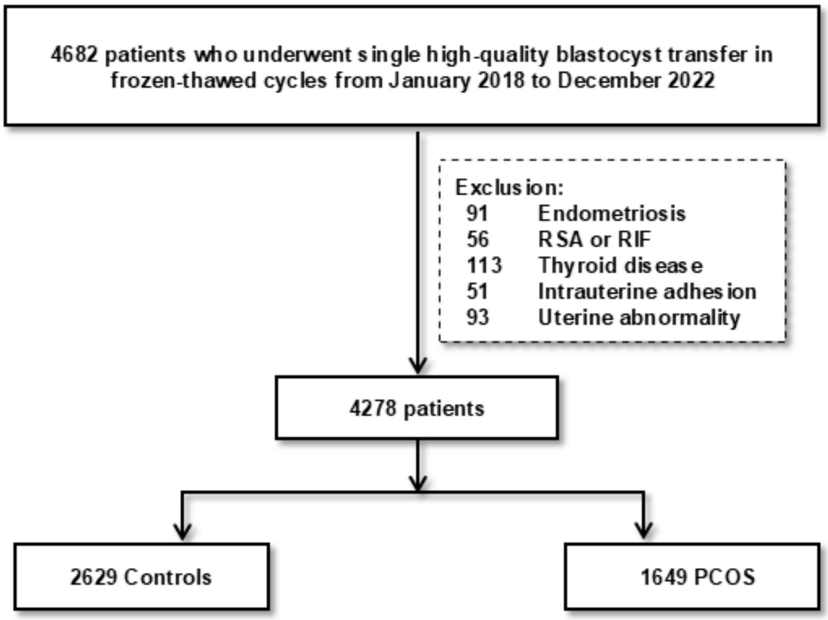

Supplementary Fig. 1: Flowchart of the study subject selection. This retrospective study analyzed 4,278 patients undergoing single high-quality blastocyst transfer. Patients with endometriosis, recurrent implantation failure (RIF), recurrent spontaneous abortion (RSA), thyroid disease, severe intrauterine adhesion, or severe uterine abnormalities were excluded. The cohort comprised 1,649 women with polycystic ovary syndrome (PCOS) and 2,629 controls after exclusion.

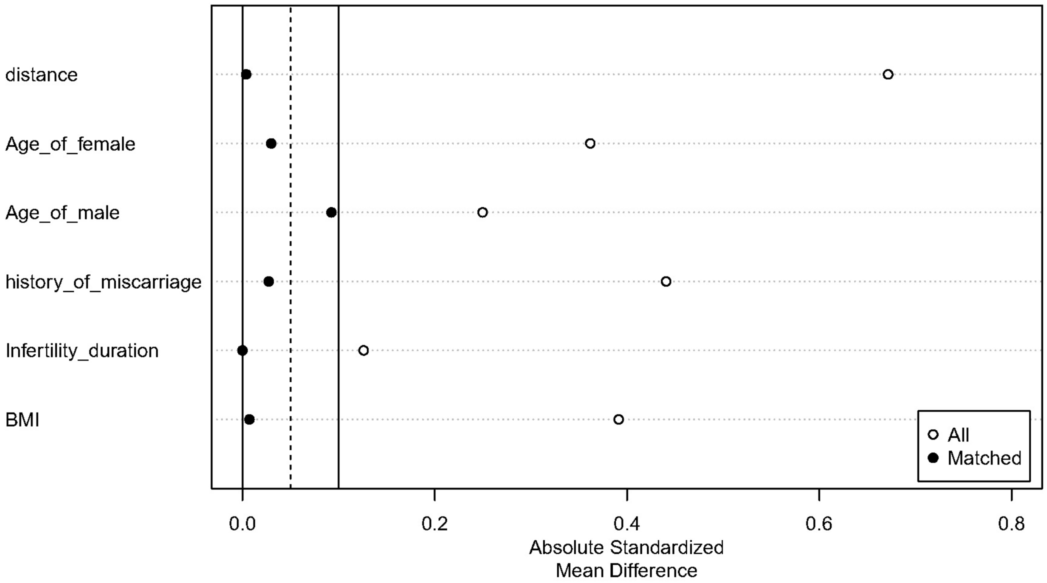

Supplementary Fig. 2: Covariate balance before and after propensity score matching. Standardized mean differences for the age of females, history of miscarriage, infertility duration, body mass index (BMI), and age of males in the PCOS and control groups, before and after matching.

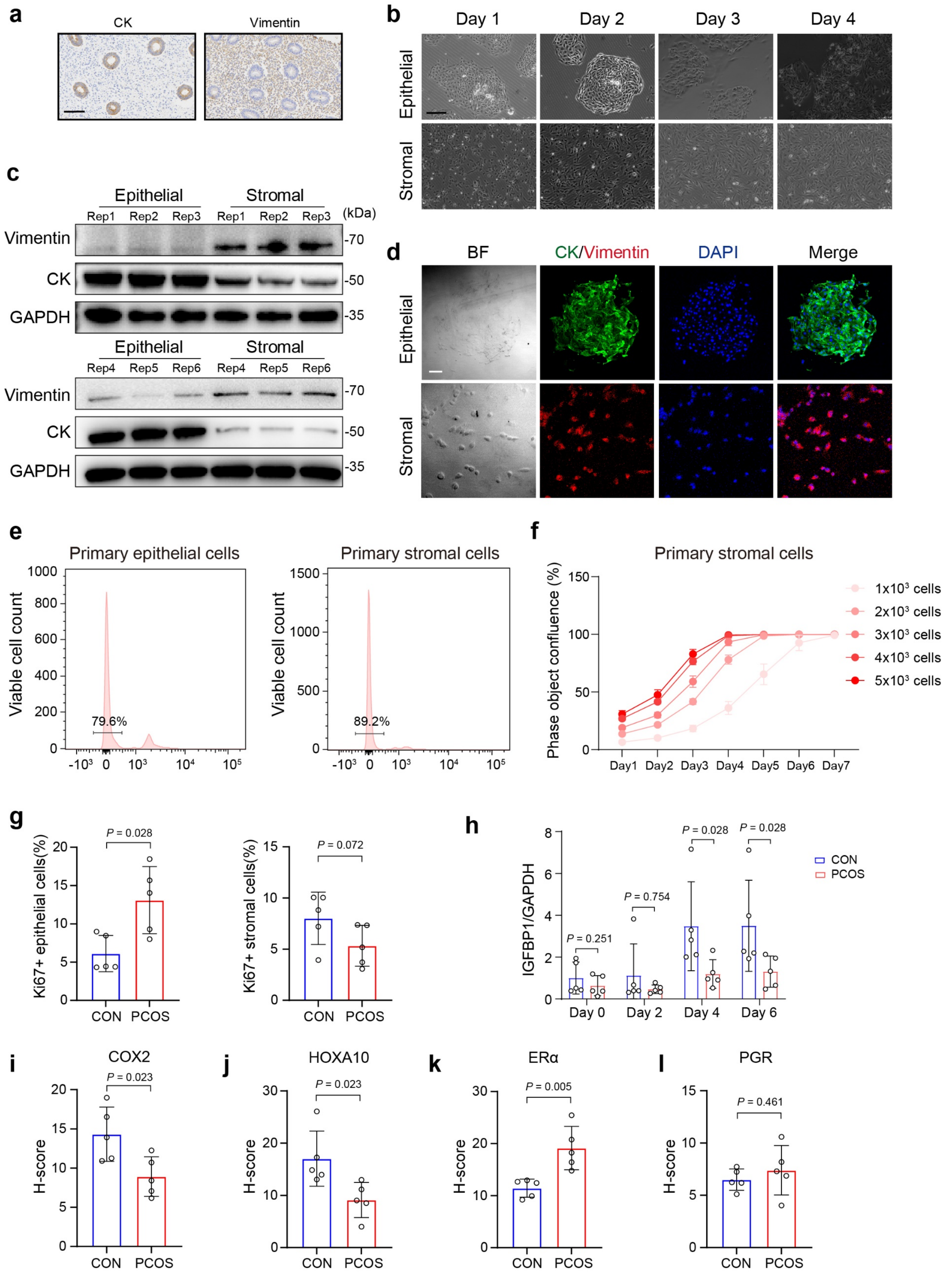

Supplementary Fig. 3: The endometrium of PCOS exhibits abnormal endometrial receptivity. a, Cytokeratin (CK) and vimentin immunohistochemistry (IHC) staining in the human endometrium. **b**, Morphology of primary endometrial epithelial and stromal cells cultured for different days. **c**, Protein levels of CK and vimentin in human primary endometrial epithelial and stromal cells (n = 6, biologically independent samples). **d**, CK and vimentin immunofluorescence (IF) staining in primary

endometrial epithelial and stromal cells. **e**, Flow cytometry plots showing 7-AAD-negative (viable) and 7-AAD-positive (non-viable) cell populations in primary epithelial cells and stromal cells. **f**, Longitudinal cell viability observation of primary endometrial stromal cells during *in vitro* culture up to 7 days. **g**, The percentage of Ki67-positive cells in mid-secretory endometrial epithelial and stromal cells (n = 5, biologically independent samples). **h**, Relative protein levels of IGFBP1 in human proliferative endometrial stromal cells during induced decidualization. (n = 5, biologically independent samples). **i-l**, Histochemical scoring assessment (H-score) of COX2, HOXA10, ERα and PGR in the mid-secretory endometrium (n = 5, biologically independent samples). For **i-l** and percentage of Ki67-positive stromal cells in **g**, P values were determined by two-tailed unpaired Student's t-test, and data are presented as means ± s.e.m. For **h** and percentage of Ki67-positive epithelial cells in **g**, P values were determined by two-tailed unpaired Mann-Whitney U rank-sum test, and data are presented as medians with interquartile ranges. For **a**-**b** and **d**, images are representative of four independent biological replicates. Scale bar: 100 μm. Source data are provided as a Source data file.

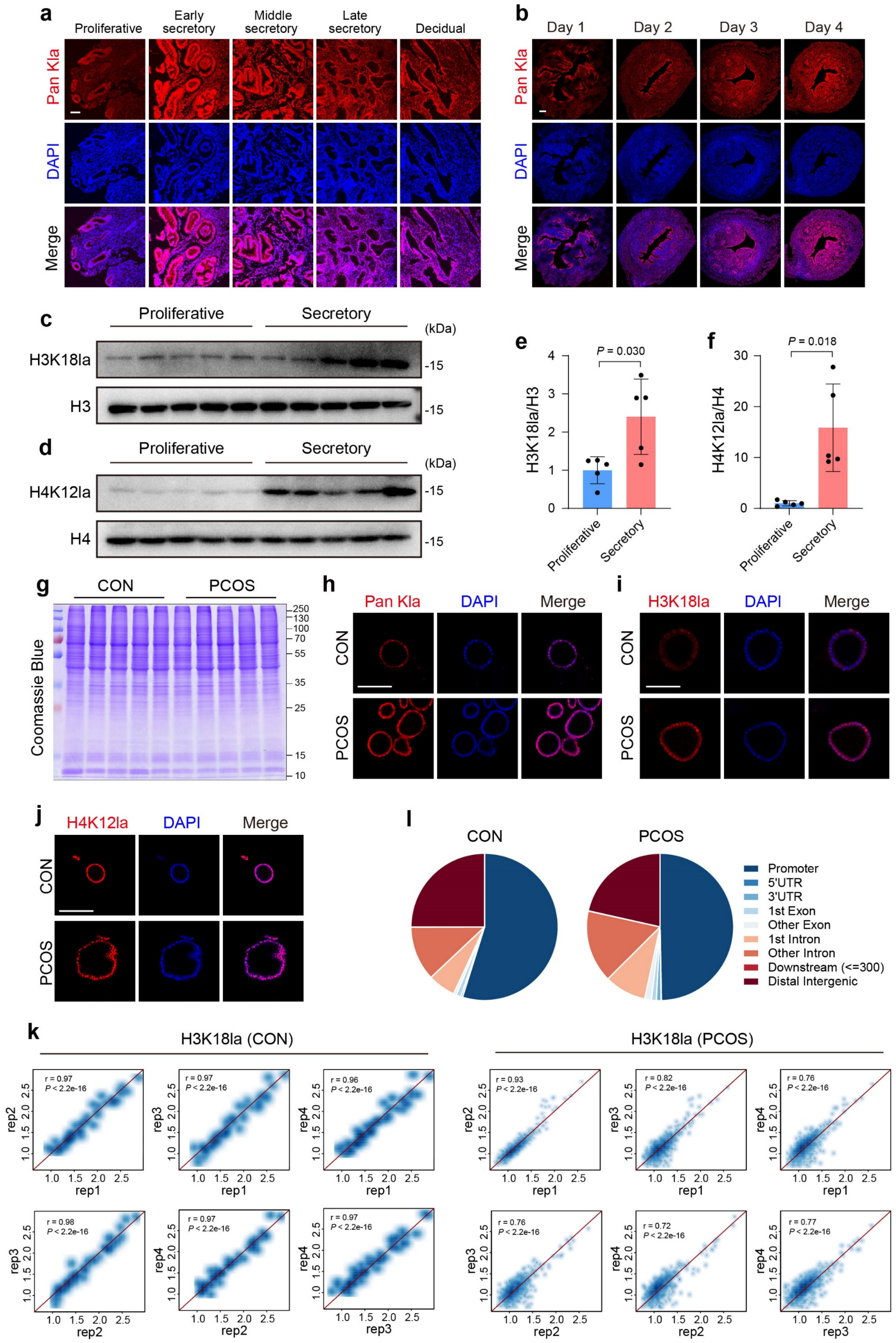

Supplementary Fig. 4: The human endometrium shows dynamic histone lysine lactylation (Kla) levels along with the menstrual cycle, as well as between PCOS and controls. a, Pan Kla IF staining in the endometrium of different menstrual stages.

b, Pan Kla IF staining in the murine uterus. **c-f**, H3K18la and H4K12la levels in proliferative and secretory endometrium (*n* = 5, biologically independent samples). **g**, Coomassie Brilliant Blue staining of proteins in the human mid-secretory endometrium. **h-j**, Pan Kla, H3K18la, and H4K12la IF staining in patient-derived endometrial organoids. **k**, Consistent H3K18la signals between biological replicates (*n* = 4, biologically independent samples). **l,** Consistent correlation of H3K18la signals between biological replicates in common peaks of endometrium between the control group and the PCOS groups (*n* = 4, biologically independent samples). For **e** and **f**, *P* values were determined by two-tailed unpaired Student's *t*-test, and data are presented as means ± s.e.m. For **a-b** and **h-j**, images are representative of four independent biological replicates. Scale bar: 100 μm. Source data are provided as a Source data file.

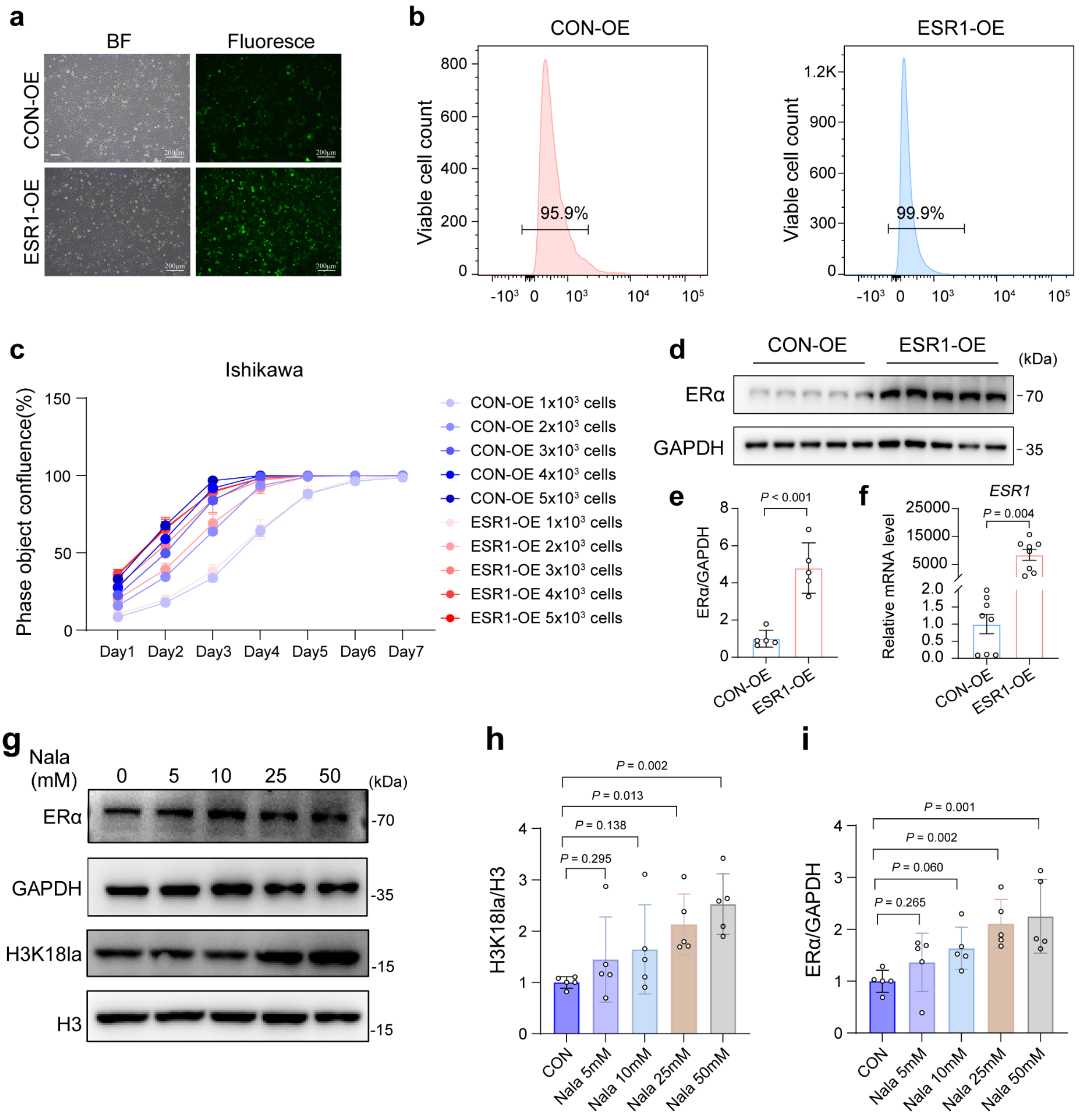

Supplementary Fig. 5: ERα and Kla upregulate each other upon their increase. a, Fluorescence imaging of Ishikawa cells 48 hours after infection. Images are representative of four independent biological replicates. Scale bar: 100 μm. **b**, Flow cytometry plots showing 7-AAD-negative (viable) and 7-AAD-positive (non-viable) cell populations in pre- and post-transfected Ishikawa cells. **c**, Longitudinal cell viability observation in CON-OE and ESR1-OE Ishikawa cells during in vitro culture up to 7 days. **d-e**, Protein levels of ERα in Ishikawa cells (n = 5, biologically independent samples). **f**, Relative mRNA levels of *ESR1* in Ishikawa cells (n = 8, biologically independent samples). **g-i**, Protein levels of H3K18la and ERα in Ishikawa cells treated with Nala at varying concentrations (5 - 50 mM) for 24 hours (n = 5, biologically independent samples). Exact P values: **e**: $P = 3.45\times10^{-4}$. For **e** and **f**, P values were determined by two-tailed unpaired Student's t-test, and data are presented as means ± s.e.m. For **h** and **i**, P values were determined by one-way ANOVA followed

by Tukey's post hoc test, and data are presented as means ± s.e.m. CON-OE, control; ESR1-OE, *ESR1* overexpression. Source data are provided as a Source data file.

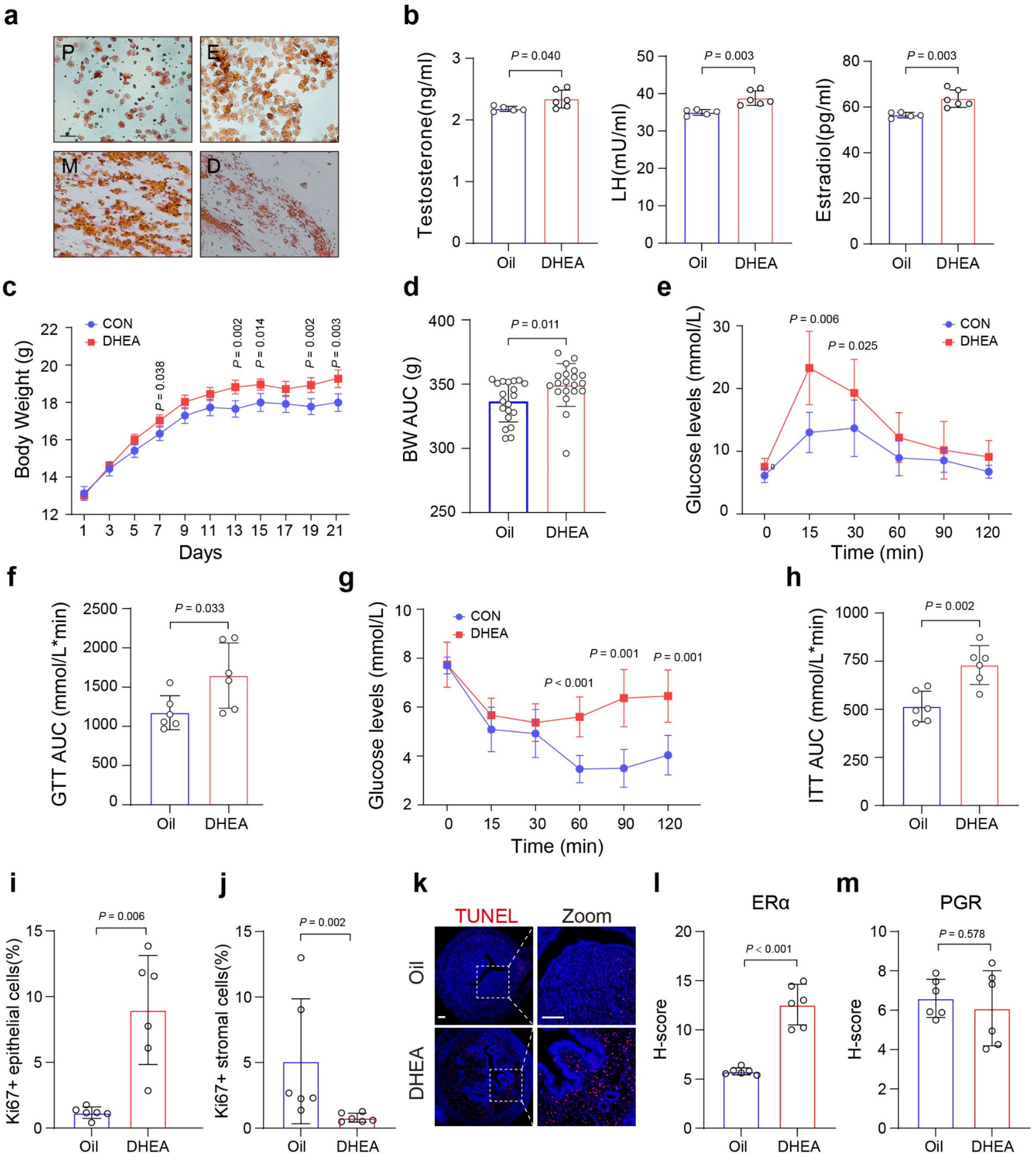

Supplementary Fig. 6: Construction and validation of PCOS model in mice. a, Representative images of vaginal smears from mouse's estrous cycle (P: proestrus, E: estrus, M: metestrus, D: diestrus). **b**, Serum testosterone (T), luteinizing hormone (LH), and estradiol (E2) levels in Oil and DHEA groups (*n* = 5 for Oil and *n* = 6 for DHEA). **c**, Changes in body weight during DHEA injection (*n* = 20, biologically independent mice). **d**, Comparison of the area under the curve (AUC) for body weight changes during drug treatment between Oil and DHEA mice (*n* = 20, biologically independent mice). **e**, Glucose tolerance tests were conducted in Oil and DHEA mice (*n* = 6,

biologically independent mice). **f**, Comparison of the AUC for glucose tolerance tests between Oil and DHEA mice (*n* = 6, biologically independent mice). **g**, Insulin tolerance tests were conducted in Oil and DHEA mice (*n* = 6, biologically independent mice). **h**, Comparison of the AUC of insulin tolerance tests between Oil and DHEA mice (*n* = 6, biologically independent mice). **i-j**, The percentage of Ki67-positive cells in endometrial epithelial and stromal cells in the murine uterus on day 4 (*n* = 6, biologically independent mice). **k**, The TUNEL assay was used to detect apoptosis in the murine uterus on day 4. **l-m**, Histochemical scoring assessment (H-score) of ERα and PGR in the murine uterus on day 4 (*n* = 6, biologically independent mice). Exact *P* values: **g**: 60 min, $P = 3.51\times10^{-4}$; **l**: $P = 3.99\times10^{-4}$. For **b**, **c**, **f**, **g**, **h**, **i**, **l**, and **m**, *P* values were determined by two-tailed unpaired Student's *t*-test, and data are presented as means ± s.e.m. For **d**, **e**, and **j**, data are presented as medians with interquartile ranges, and *P* values were determined by two-tailed unpaired Mann-Whitney *U* rank-sum test. For **a** and **k**, images are representative of four independent biological replicates. Scale bar: 100 μm. Source data are provided as a Source data file.

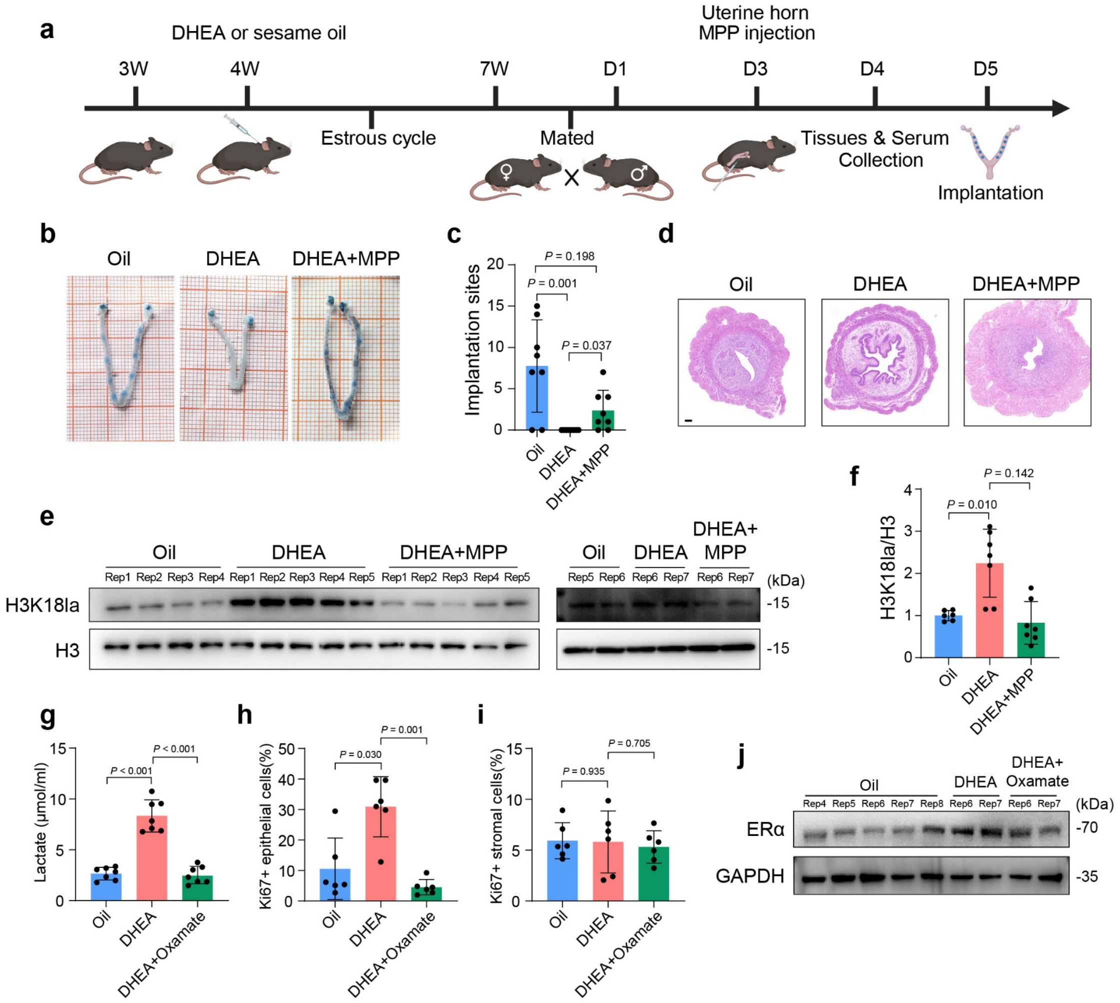

Supplementary Fig. 7: Inhibition of ERα or lactylation rescues impaired endometrial receptivity in PCOS.

a, Schematic diagram of PCOS model mice construction and MPP intervention. Created in BioRender. Hongying, S. (2025) https://BioRender.com/gms0kcg. **b**, Representative images of implantation sites (ISs) on day 5 in Oil, DHEA, and DHEA+MPP groups (8.14 μg/kg MPP treatment). **c**, The number of ISs on day 5 in Oil, DHEA, and DHEA+MPP groups (n = 8, biologically independent mice). **d**, Hematoxylin and eosin (H&E) staining of uterine morphology on day 4 in Oil, DHEA, and DHEA+MPP groups. **e-f**, Uterine H3K18la levels on day 4 in Oil (n = 6), DHEA (n = 7), and DHEA+MPP (n = 7) groups. Images presented are representative of four independent biological replicates. Scale bar: 100 μm. **g**, Lactate levels in the murine uterus on day 4 (*n* = 7, biologically independent mice). **h-i**, The percentage of Ki67-positive cells in endometrial epithelial and stromal cells in the murine uterus on day 4 (*n* = 6, biologically independent mice). **j**, Protein levels of ERα in the murine uterus on day 4

($n = 8$ for Oil, $n = 7$ for DHEA and DHEA+Oxamate). Exact P values: **g**: Oil vs DHEA, $P = 1.41\times10^{-8}$; DHEA vs DHEA+Oxamate, $P = 9.81\times10^{-9}$. For **f**, **g**, and **i**, P values were determined by one-way ANOVA followed by Tukey's post hoc test, and data are presented as means ± s.e.m. For **c** and **h**, data are presented as medians with interquartile ranges, and P values were determined by Kruskal-Wallis test followed by Dunn's post hoc test. Source data are provided as a Source data file.

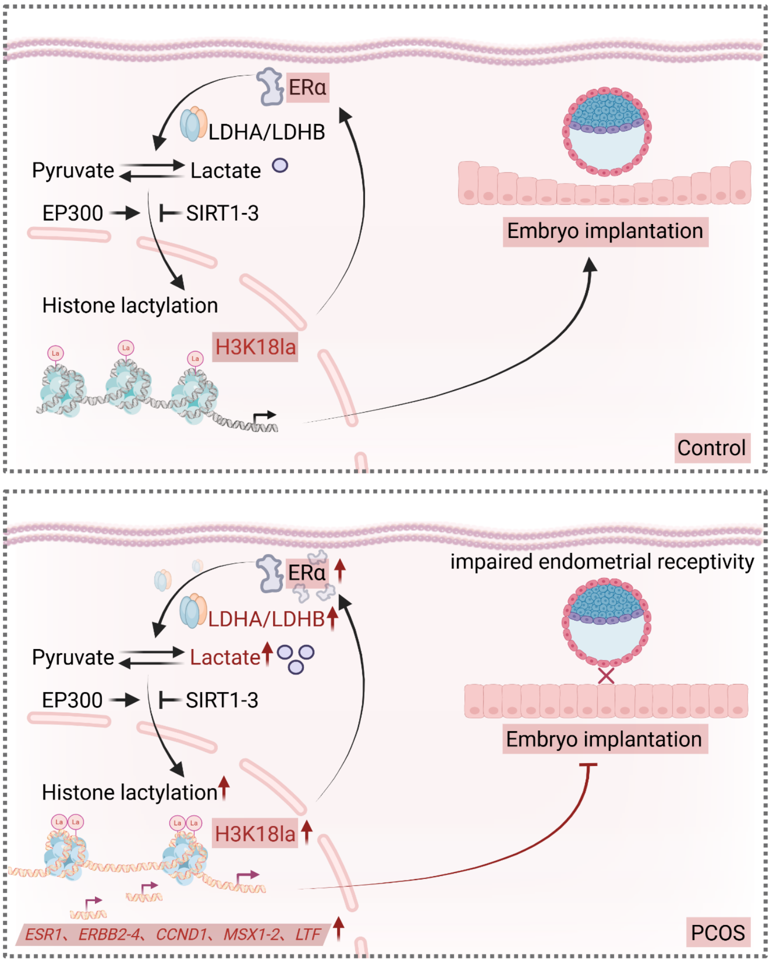

Supplementary Fig. 8: Diagrammatic representation of impaired H3K18la function in the uterus during the establishment of endometrial receptivity in women with PCOS. The endometrium of women with PCOS exhibited impaired receptivity along with elevated ERα and H3K18la levels. ERα overexpression increases H3K18la via LDHA/LDHB, and increased H3K18la could also promote ERα expression. Created in BioRender. Hongying, S. (2025)https://BioRender.com/gn1p1bz.

Supplementary Table 1. Analysis of the basic data of the two groups before or after matching in frozen-thawed embryo transfer cycles

FET cycles	Before PSM			After PSM		
Item	Control group (n=2629)	PCOS group (n=1649)	*P* value	Control group (n=1406)	PCOS group (n=1406)	*P* value
Age of female (year)	32.4±3.7	31.0±3.8	1.60E-30	31.3±3.6	31.5±3.8	0.403
Age of male (year)	33.4±4.8	32.2±5.0	7.00E-16	32.3±4.6	32.8±5.0	0.011
BMI (kg/m^2)	22.5±3.3	24.2±4.1	7.00E-45	23.3±3.6	23.8±4.0	4.70E-04
Infertility type (%)			1.30E-47			0.004
Primary infertility	1191 (45.3)	1122 (68.0)		823 (58.5)	899 (63.9)	
Secondary infertility	1438 (54.7)	527 (32.0)		583 (41.5)	507 (36.1)	
Infertility duration(year)	2 [1, 4]	3 [2, 4]	2.40E-06	3 [2, 4]	3 [2, 4]	0.215
History of miscarriage (%)			3.80E-37			0.518
No	1222 (46.5)	444 (26.9)		458 (32.6)	441 (31.4)	
Yes	1407 (53.5)	1205 (73.1)		948 (67.4)	965 (68.6)	
Basal FSH(U/L)	5.50 [4.26, 6.76]	5.51 [4.53, 6.66]	0.479	5.48 [4.20, 6.68]	5.59 [4.55, 6.69]	0.035
Basal LH(U/L)	2.96 [1.96, 4.34]	5.22 [3.40, 7.72]	7.20E-133	2.91 [1.91, 4.21]	5.19 [3.41, 7.58]	9.50E-114
Basal E2(pmol/L)	149 [118, 192]	160 [128, 208]	1.20E-12	152 [118, 193]	159 [126, 208]	1.90E-05

FET cycles	Before PSM			After PSM		
Item	Control group (n=2629)	PCOS group (n=1649)	*P* value	Control group (n=1406)	PCOS group (n=1406)	*P* value
Basal P (nmol/L)	1.04 [0.81, 1.34]	1.03 [0.78, 1.38]	0.775	1.04 [0.79, 1.35]	1.03 [0.78, 1.39]	0.936
Basal A (nmol/L)	6.23 [4.52, 8.44]	8.96 [6.41, 12.60]	1.10E-64	6.34 [4.66, 8.72]	8.68 [6.35, 12.20]	9.10E-61
Basal T (μg/L)	0.69 [0.69, 0.93]	0.73 [0.69, 1.17]	1.30E-36	0.69 [0.69, 0.93]	0.71 [0.69, 1.14]	2.00E-10
Basal PRL (nmol/L)	11.80 [8.62, 16.70]	11.70 [8.68, 16.30]	0.941	12.10 [8.71, 16.91]	11.80 [8.68, 16.30]	0.472
AMH (μg/L)	3.06 [1.91, 4.71]	6.92 [4.46,10.52]	1.70E-177	3.13 [1.97, 4.78]	6.93 [4.49, 10.51]	6.70E-172
AFC	11 [8, 14]	19 [14, 24.]	1.30E-242	11 [8, 15]	19 [14, 24]	1.60E-164
hCG injection day						
E2 (pmol/L)	9361 [6057, 13249]	11013 [6432, 17177]	5.70E-13	9253 [5940, 13103]	11150 [6445, 17265]	2.70E-14
LH (U/mL)	0.96 [0.53, 1.92]	1.69 [0.86, 3.34]	4.10E-57	0.97 [0.52, 1.89]	1.67 [0.83, 3.24]	4.50E-43
P (nmol/L)	2.29 [1.60, 3.21]	1.92 [1.31,2.92]	2.20E-17	2.22 [1.56, 3.15]	1.97 [1.33, 2.97]	1.30E-07
Endometrial thickness (mm)	10.0 [9.0, 11.0]	10.0 [9.0, 10.7]	3.80E-06	10.0 [9.0, 11.0]	10.0 [9.0, 10.8]	2.10E-08
Insemination method (%)			0.299			0.142
IVF	1963 (74.7)	1247 (75.6)		1036 (73.7)	1047 (74.5)	

FET cycles	Before PSM			After PSM		
Item	Control group (n=2629)	PCOS group (n=1649)	*P* value	Control group (n=1406)	PCOS group (n=1406)	*P* value
ICSI	592 (22.5)	368 (22.3)		318 (22.6)	325 (23.1)	
IVF+ICSI	74 (2.8)	34 (2.1)		52 (3.7)	34 (2.4)	
Endometrial preparation protocol			4.90E-178			3.90E-115
Natural Cycle	1488 (56.6)	244 (14.8)		781 (55.5)	218 (15.5)	
Artificial Cycle	929 (35.3)	1299 (78.8)		515 (36.6)	1094 (77.8)	
Ovarian stimulation cycle	212 (8.1)	106 (6.4)		110 (7.8)	94 (6.7)	

Notes: FET: frozen-thawed embryo transfer; PCOS: polycystic ovary syndrome; BMI: body mass index; FSH: follicle-stimulating hormone; LH: luteinizing hormone; E2: estradiol; P: progesterone; A: androstenedione; T: testosterone; PRL: prolactin; AMH: anti-mullerian hormone; AFC: antral follicle count; hCG: human chorionic hormone; positive number/total number in brackets.

Continuous variables were tested for normality using Shapiro–Wilk test. Normally distributed data are presented as Means ± SD and compared using two-tailed unpaired Student's *t* test. Non-normally distributed data are presented as medians [Q1, Q3] and compared using two-tailed unpaired Mann–Whitney *U* rank-sum test. Categorical variables are summarized as numbers (percentages) and compared using chi-square test. All statistical analyses were performed in R.

Supplementary Table 2. Analysis of baseline data of endometrial samples between the two groups

Item	Control group (n =25)	PCOS group (n =14)	*P* value
Age of female (year)	32.5±3.1	31.6±3.7	0.396
BMI (kg/m^2)	21.1±3.0	24.4±3.3	0.004
Infertility duration (year)	3.3±1.8	3.8±1.3	0.400
SBP (mmHg)	121.80±9.97	116.50±7.63	0.093
DBP (mmHg)	77.96±11.96	73.14±9.27	0.201
FSH (U/L)	6.40±1.75	5.01±1.31	0.016
LH (U/L)	4.09±1.61	6.50±3.74	0.008
E2 (pmol/L)	150 [118, 182]	108 [91, 185]	0.202
T (μg/L)	0.69 [0.69, 0.79]	0.69 [0.69, 0.87]	0.883
A (nmol/L)	5.82±3.06	8.92±5.10	0.039
PRL (nmol/L)	13.39±5.35	15.98±6.06	0.192
PRG (nmol/L)	2.46±3.85	4.16±10.24	0.471
AFC	14.52±5.59	29.57±9.48	4.00E-05
AMH (μg/L)	3.19±1.78	9.38±4.77	3.00E-04
GLU-0 (mmol/L)	5.13±0.47	5.16±0.59	0.873
ALT (U/L)	11 [10, 13]	16 [9, 35]	0.126
AST (U/L)	17 [15, 19]	18 [16, 26]	0.245
TBil (μmol/L)	13.74±5.46	10.25±2.97	0.034
DBil (μmol/L)	1.38±0.55	0.82±0.40	0.003
Urea (mmol/L)	4.28±0.96	4.68±1.65	0.341
Cr (μmol/L)	66.60±9.91	68.07±7.33	0.631
UA (μmol/L)	273.40±62.52	320.71±75.01	0.042
TC (mmol/L)	4.47±0.69	5.29±1.14	0.008
TG (mmol/L)	0.84±0.34	1.74±1.16	0.013
HDL (mmol/L)	1.44±0.27	1.23±0.25	0.023
LDL (mmol/L)	2.52±0.61	3.30±0.98	0.004
TSH (μIU/ml)	2.38±2.77	2.39±0.97	0.991

Note: SBP: systolic blood pressure; DBP: diastolic blood pressure; TSH: follicle stimulating hormone; LH: luteinizing hormone; E2: estradiol; T: testosterone; A:

androstenedione; PRL: prolactin; P: progesterone; AFC: antral follicle count; AMH: anti-müllerian hormone; FBG: fasting blood glucose; ALT: alanine aminotransferase; AST: aspartate aminotransferase; TBil: total bilirubin; DBil: direct bilirubin; Cr: creatinine; UA: uric acid; TC: total cholesterol; TG: triglycerides; HDL: high-density lipoprotein cholesterol; LDL: low-density lipoprotein cholesterol; TSH: thyroid stimulating hormone.

Continuous variables were tested for normality using Shapiro–Wilk test. Normally distributed data are presented as Means ± SD and compared using two-tailed unpaired Student's *t* test. Non-normally distributed data are presented as medians [Q1, Q3] and compared using two-tailed unpaired Mann–Whitney *U* rank-sum test. All statistical analyses were performed in R.

Supplementary Table 3. Information on antibodies.

Antibodies	Source	Identifity	Dilution	Species Reactivity
Rabbit anti-IGFBP1 antibody	CST	31025T	1:1000	Human
Rabbit anti-Progesterone Receptor antibody	CST	8757S	1:1000	Human
Rabbit anti-Estrogen Receptor alpha antibody	Abcam	ab32063	1:1000	Mouse, Rat, Human
Rabbit anti-COX2 antibody	Abcam	ab179800	1:200	Mouse, Rat, Human
Goat anti-HOXA10 antibody	Abcam	ab191470	1:100	Human, Mouse, Rat, Rabbit, Horse, Chicken, Cow, Pig, Chimpanzee, Monkey, Gorilla
Rabbit anti-Ki67 antibody	Abcam	ab16667	1:200	Mouse, Rat, Human
Rabbit anti-MUC1antibody	Abcam	ab109185	1:200	Mouse, Rat, Human
Rabbit anti-Cytokeratin antibody	Abcam	ab52625	1:10000	Mouse, Human
Mouse anti-Vimentin antibody	Abcam	ab8978	1:1000	Mouse, Rat, Human, Pig, Zebrafish
Rabbit anti-pan Kla antibody	PTM BIO	PTM-1401	IF:1:50, WB:1:1000	All species
Rabbit anti-H3K18la antibody ChIP Grade	PTM BIO	PTM-1427RM	Cut&tag:1:50	Human, Mouse
Rabbit anti-H3K18la antibody	PTM BIO	PTM1406RM	1:1000	Human, Mouse, Rat
Rabbit anti-H4K12la antibody	PTM BIO	PTM1411RM	1:1000	Human, Mouse, Rat
Rabbit anti-Histone H4 antibody	PTM BIO	PTM1015RM	1:2000	Human, Mouse, Rat, Monkey, Pig, S.cerevisiae
Rabbit anti-Histone H3 antibody	PTM BIO	PTM1002RM	1:2500	Human, Mouse, Rat, Rice
Mouse anti-ERα Antibody	Immunoway	YM0252	1:200	Human
Rabbit anti-LDHB antibody	Proteintech	14824-1-AP	1:10000	Human, Mouse, Rat, Pig, Chicken, Bovine, Sheep
Rabbit anti-LDHA antibody	Proteintech	19987-1-AP	1:5000	Human, Mouse, Rat, Rabbit, Chicken, Goat
Rabbit anti-GAPDH antibody	Abcam	ab181602	1:10000	Mouse, Rat, Chicken, Human, Zebrafish, African green monkey, Xenopus tropicalis
Mouse anti -beta ACTIN antibody	Abcam	ab6276	1:10000	Mouse, Rat, Cow, Dog, Human, African green monkey, Chinese hamster
HRP-conjugated Affinipure Donkey anti-Goat IgG(H+L)	Beyotime	A0181	1:1000	Unspecified reactive species
HRP-conjugated Affinipure Goat Anti-Rabbit IgG(H+L)	Beyotime	A0208	1:1000	Unspecified reactive species
HRP-conjugated Affinipure Goat Anti-Mouse IgG(H+L)	Beyotime	A0216	1:1000	Unspecified reactive species
Phalloidin-iFluor 555 Reagent	Abcam	ab176756	1:200	Unspecified reactive species
Phalloidin-iFluor 488 Reagent	Abcam	ab176753	1:200	Unspecified reactive species

Supplementary Table 4. Information on qRT-PCR primers.

Gene	Forward （5' to 3'）	Reverse （3' to 5'）	GenBank accession numbers	Amplicon Size	R2	Amplificatic efficiency	Slope	Primer Concentrations
human-GAPDH	GGAGCGAGATCCCTCCAAAAT	GGCTGTTGTCATACTTCTCATGG	AK299972.1	197bp	0.998	96.048	-3.420	10 μM
human-18S	GTAACCCGTTGAACCCCATT	CCATCCAATCGGTAGTAGCG	8UJ9_S2	151bp	0.999	94.435	-3.463	10 μM
human-ACTB	TGCCCATCTACGAGGGGTAT	CTTAATGTCACGCACGATTTCC	PQ040393.1	152bp	0.999	94.675	-3.457	10 μM
human-EP300	GCTTCAGACAAGTCTTGGCAT	ACTACCAGATCGCAGCAATTC	BC053889.1	79bp	0.993	103.720	-3.236	10 μM
human-LDHA	TTGACCTACGTGGCTTGGAAG	GGTAACGGAATCGGGCTGAAT	BC051361.1	91bp	0.993	106.879	-3.167	10 μM
human-LDHB	TCTGTGACCGCCAATTCTAAGA	GCACCAGATTGAGCCGACTC	EU919185.1	82bp	0.993	103.949	-3.231	10 μM
human-SIRT1	AAGTTGACTGTGAAGCTGTACG	TGCTACTGGTCTTACTTTGAGGG	JQ768366.1	218 bp	0.996	96.389	-3.412	10 μM
human-SIRT2	ATCCACCGGCCTCTATGACAA	CGCATGAAGTAGTGACAGATGG	KF032391.1	162bp	0.997	109.280	-3.118	10 μM
human-SIRT3	CCCCAAGCCCTTTTTCACTTT	CGACACTCTCTCAAGCCCA	AF083108.2	148bp	0.995	91.038	-3.557	10 μM
human-PRL	ATCATCTGGTCACGGAAGTACG	GGTTTGCTCCTCAATCTCTACAG	BC088370.1	83bp	0.990	94.216	-3.469	10 μM
human-IGFBP1	TTGGGACGCCATCAGTACCTA	TTGGCTAAACTCTCTACGACTCT	FJ795026.1	114bp	0.995	109.335	-3.117	10 μM
human-ESR1	CCCACTCAACAGCGTGTCTC	CGTCGATTATCTGAATTTGGCCT	LC516420.1	180bp	0.999	102.598	-3.261	10 μM
human-MSX1	ACACAAGACGAACCGTAAGCC	CACATGGGCCGTGTAGAGTC	HM213930.1	382bp	0.993	96.266	-3.416	10 μM
human-MSX2	TGCAGAGCGTGCAGAGTTC	GGCAGCATAGGTTTTGCAGC	AH004951.2	144bp	0.997	98.052	-3.369	10 μM
human-LTF	CCCAGGAACCGTACTTCAGC	GTGCCACAACGGCATGAGA	KT006756.1	219bp	0.992	104.852	-3.211	10 μM
human-PGR	CCCAGCATGTCGCCTTAGAAA	AGTGCTCTCACAACTCTGACTT	AY382151.1	96bp	0.999	109.888	-3.106	10 μM
human-AREG	GTGGTGCTGTCGCTCTTGATA	CCCCAGAAAATGGTTCACGCT	BT019866.1	97bp	0.994	108.596	-3.132	10 μM
human-IHH	TCCGTCAAGTCCGAGCACT	GTCCTGAGTCTCGATGACCTG	BC136588.1	228bp	0.999	90.031	-3.586	10 μM
mouse-Gapdh	TGGCCTTCCGTGTTCCTAC	GAGTTGCTGTTGAAGTCGCA	OX390160.1	178bp	0.990	105.802	-3.190	10 μM
mouse-Actb	GTGACGTTGACATCCGTAAAGA	GCCGGACTCATCGTACTCC	BC138614.1	245bp	0.997	109.838	-3.107	10 μM
mouse-Tbp	ACCGTGAATCTTGGCTGTAAAC	GCAGCAAATCGCTTGGGATTA	BC016476.1	86bp	0.992	109.964	-3.104	10 μM
mouse-36b4	CTCACTGAGATTCGGGATATG	CTCCCACCTTGTCTCCAGTC	BC011291.1	223bp	0.993	103.523	-3.240	10 μM
mouse-Esr1	TCTGCCAAGGAGACTCGCTACT	GGTGCATTGGTTTGTAGCTGGAC	LC260511.1	153bp	0.996	104.478	-3.219	10 μM
mouse-Msx1	GCACAAGACCAACCGCAAG	CGCTCGGCAATAGACAGGT	BC016426.1	102bp	0.994	106.096	-3.184	10 μM
mouse-Msx2	CTAAAGGCGGTGACTTGTTTTCG	CGGCTTCTTGTCGGACATGAG	BC141132.1	161bp	0.990	102.222	-3.270	10 μM
mouse-Ltf	GTCTGCCATTGGCTTTGTGAGG	CCTTTGAGGCTATCACATCCTGC	BC006904.2	122bp	0.992	108.303	-3.138	10 μM
