# Supplementary material for: Women with polycystic ovary syndrome exhibit impaired endometrial receptivity with excessive ERα and histone lactylation
Source: Nat Commun. 2026 Jan 21;17:1739. doi: 10.1038/s41467-026-68441-0 (PMC12913789; doi:10.1038/s41467-026-68441-0)
Supplement: Supplementary file 2 — Description Of Additional Supplementary File [file 41467_2026_68441_MOESM2_ESM.pdf]

Description of Additional supplementary files

Supplementary Data 1:

This section presents methodological validations and analytical results. It details the amplification efficiency of real-time PCR (qRT-PCR) primers (for human and mouse reference/target genes) and includes negative controls for qRT-PCR, Western blot (WB), immunohistochemistry (IHC), and immunofluorescence (IF). Additionally, it shows relative mRNA levels normalized to ACTB (human) and Gapdh (mouse), and assesses the effects of BMI on endometrial ERα and H3K18la levels in controls and PCOS patients.
