# Supplementary material for: Women with polycystic ovary syndrome exhibit impaired endometrial receptivity with excessive ERα and histone lactylation
Source: Nat Commun. 2026 Jan 21;17:1739. doi: 10.1038/s41467-026-68441-0 (PMC12913789; doi:10.1038/s41467-026-68441-0)
Supplement: Supplementary file 3 — Supplementary Data 1 [file 41467_2026_68441_MOESM3_ESM.pdf]

Supplementary Data 1

Figures

Supplementary Data Figure 1. The amplification efficiency of human reference genes.

Supplementary Data Figure 2. The amplification efficiency of mouse reference genes.

Supplementary Data Figure 3. The amplification efficiency of human target genes.

Supplementary Data Figure 4. The amplification efficiency of mouse target genes.

Supplementary Data Figure 5. Relative mRNA levels normalized to *ACTB* as the human reference gene and to *Gapdh* as the mouse reference gene.

Supplementary Data Figure 6. Negative controls of qRT-PCR.

Supplementary Data Figure 7. Negative controls of Western blots (WB).

Supplementary Data Figure 8. Negative controls of immunohistochemistry (IHC) and immunofluorescent (IF) staining.

Supplementary Data Figure 9. Elevated endometrial ERα and H3K18la levels are PCOS-dependent but not BMI-dependent.

Supplementary Data Figure 10. Endometrial ERα and H3K18la levels are consistently upregulated in PCOS compared to controls with matched BMIs.

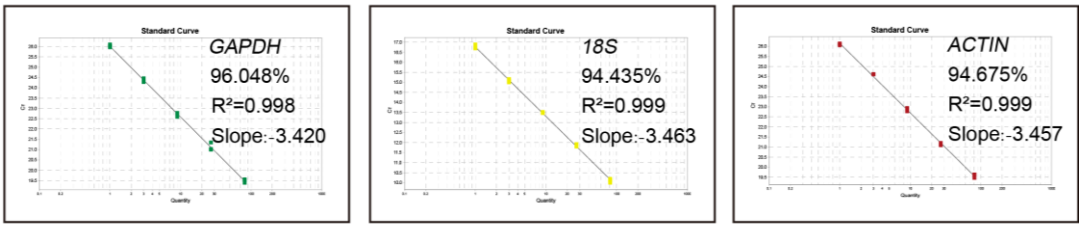

Supplementary Data Figure 1: The amplification efficiency of human reference genes. Validation of human reference genes (*GAPDH*, *18S*, *ACTB*) with amplification efficiencies of 90–110% and $R^2 \geq 0.99$ using standard curve analysis.

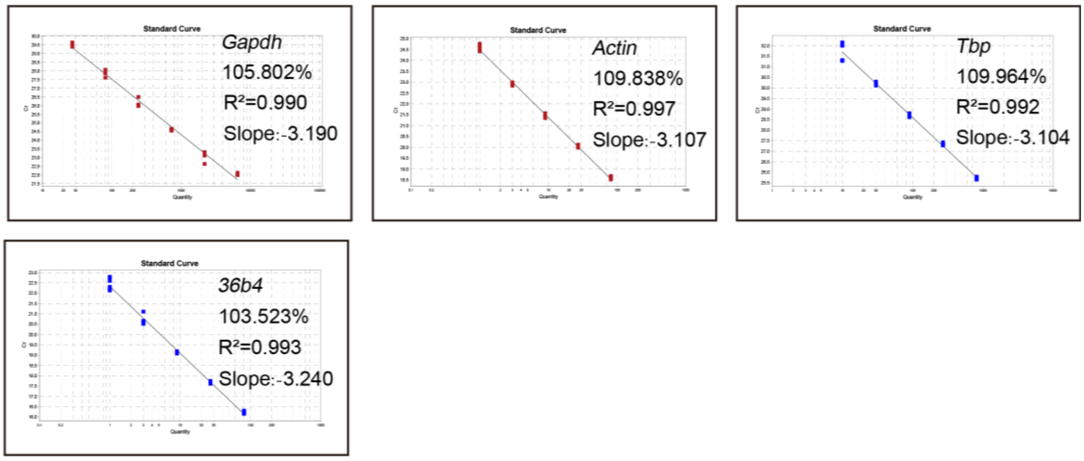

Supplementary Data Figure 2: The amplification efficiency of mouse reference genes. Validation of mouse reference genes (*Gapdh*, *Actb*, *Tbp* and *36b4*) with amplification efficiencies of 90–110% and $R^2 \geq 0.99$ using standard curve analysis.

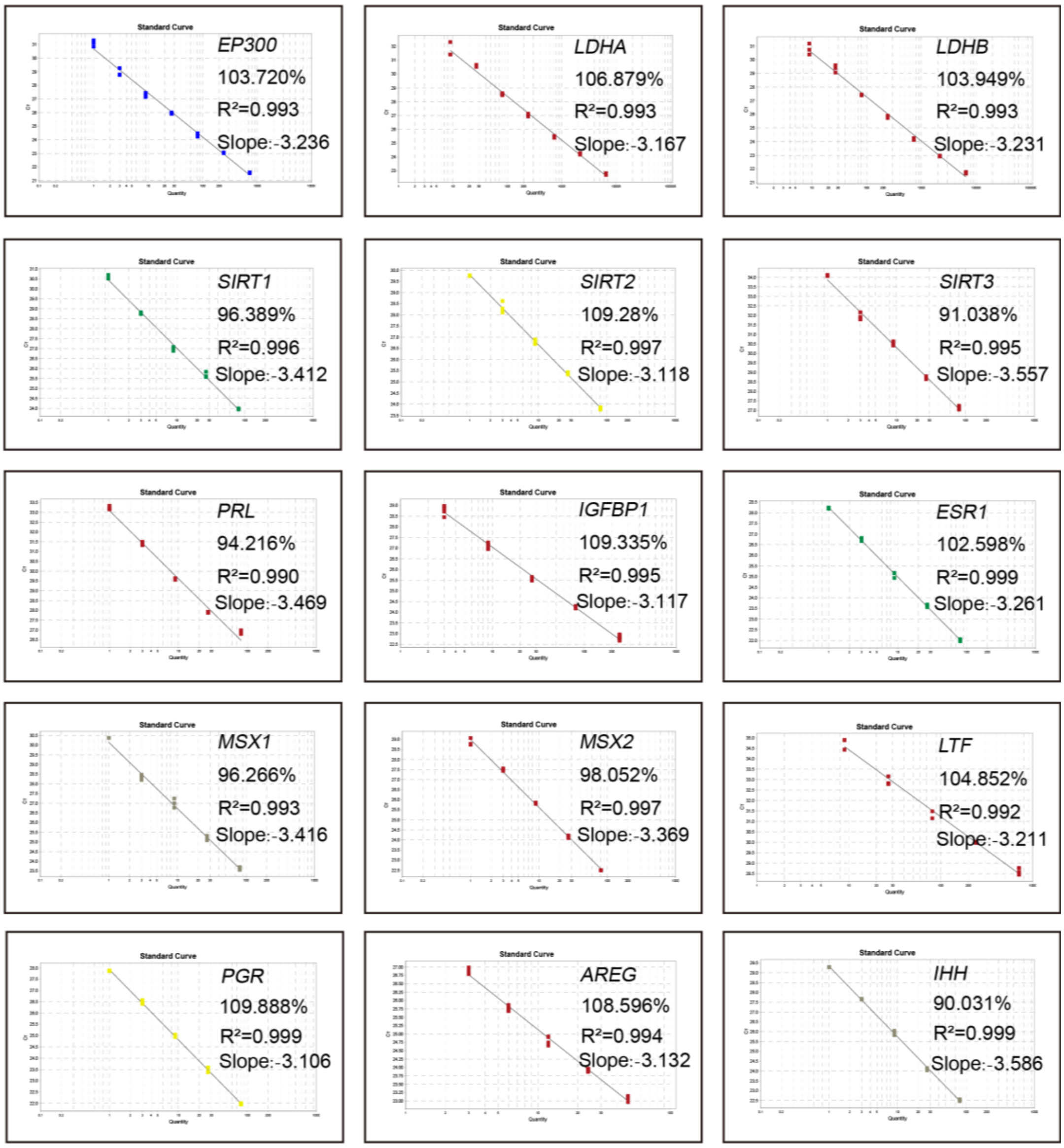

Supplementary Data Figure 3. The amplification efficiency of human target genes. Validation of human target genes (*EP300*, *LDHA*, *LDHB*, *SIRT1*, *SIRT2*, *SIRT3*, *PRL*, *IGFBP1*, *ESR1*, *MSX1*, *MSX2*, *LTF*, *PGR*, *AREG*, *IHH*) with amplification efficiencies of 90–110% and $R^2 \geq 0.99$ using standard curve analysis.

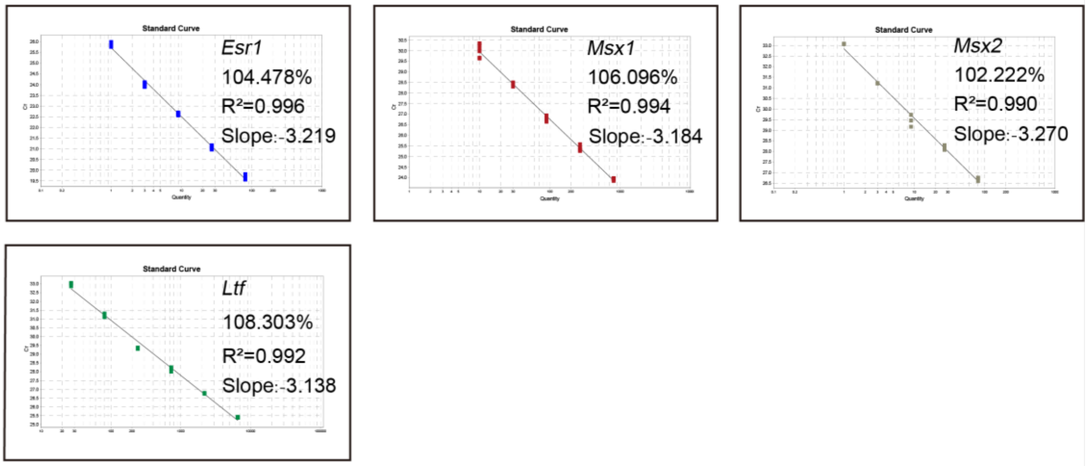

Supplementary Data Figure 4: The amplification efficiency of mouse target genes. Validation of mouse target genes (*Esr1*, *Msx1*, *Msx2*, *Ltf*) with amplification efficiencies of 90–110% and $R^2 \geq 0.99$ using standard curve analysis.

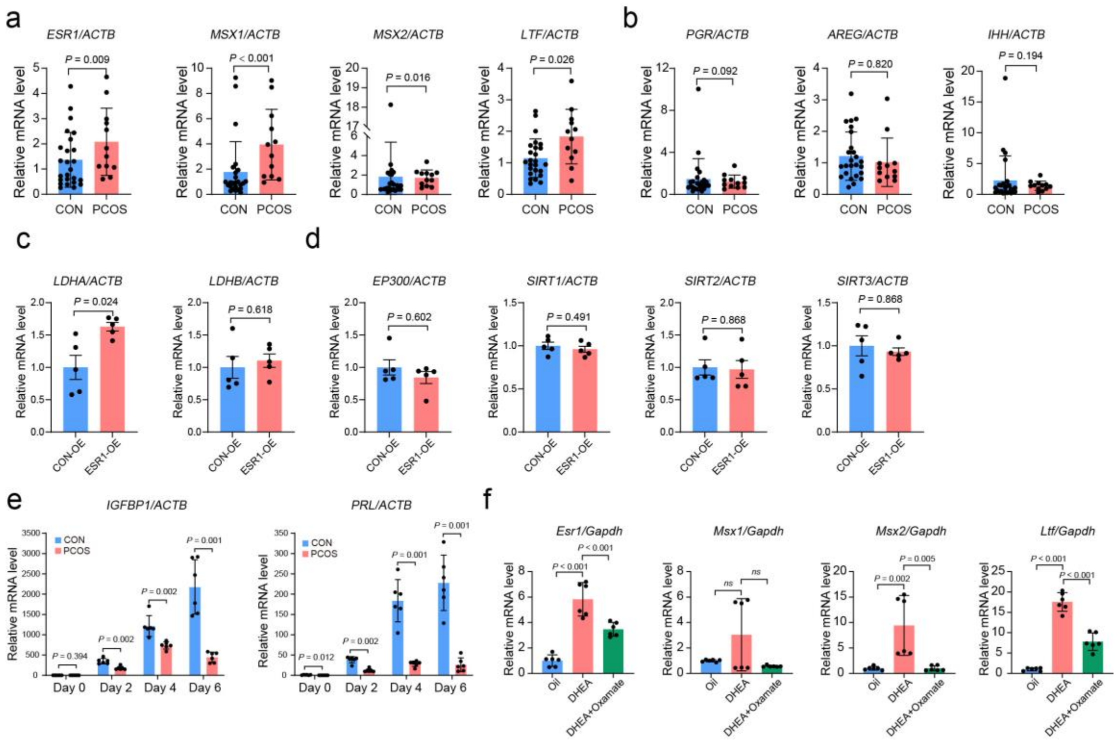

Supplementary Data Figure 5: Relative mRNA levels normalized to *ACTB* as the human reference gene and to *Gapdh* as the mouse reference gene. a, Relative mRNA levels of *ESR1*, *MSX1*, *MSX2*, and *LTF* normalized to *ACTB* in the human mid-secretory endometrium ($n = 25$ for CON and $n = 12$ for PCOS, biologically independent samples). **b**, Relative mRNA levels of *PGR*, *AREG*, and *IHH* normalized to *ACTB* in the human mid-secretory endometrium ($n = 25$ for CON and $n = 12$ for PCOS, biologically independent samples). **c**, Relative mRNA levels of *LDHA* and *LDHB* normalized to *ACTB* in Ishikawa cells ($n = 5$, biologically independent samples). **d**, Relative mRNA levels of *EP300*, *SIRT1*, *SIRT2*, and *SIRT3* normalized to *ACTB* in Ishikawa cells ($n = 5$, biologically independent samples). **e**, Relative mRNA levels of *IGFBP1* and *PRL* normalized to *ACTB* in human primary endometrial stromal cells during induced decidualization ($n = 6$, biologically independent samples). **f**, Relative mRNA levels of *Esr1*, *Msx1*, *Msx2*, and *Ltf* normalized to *Gapdh* in the murine uterus on day 4 ($n = 6$, biologically independent samples).

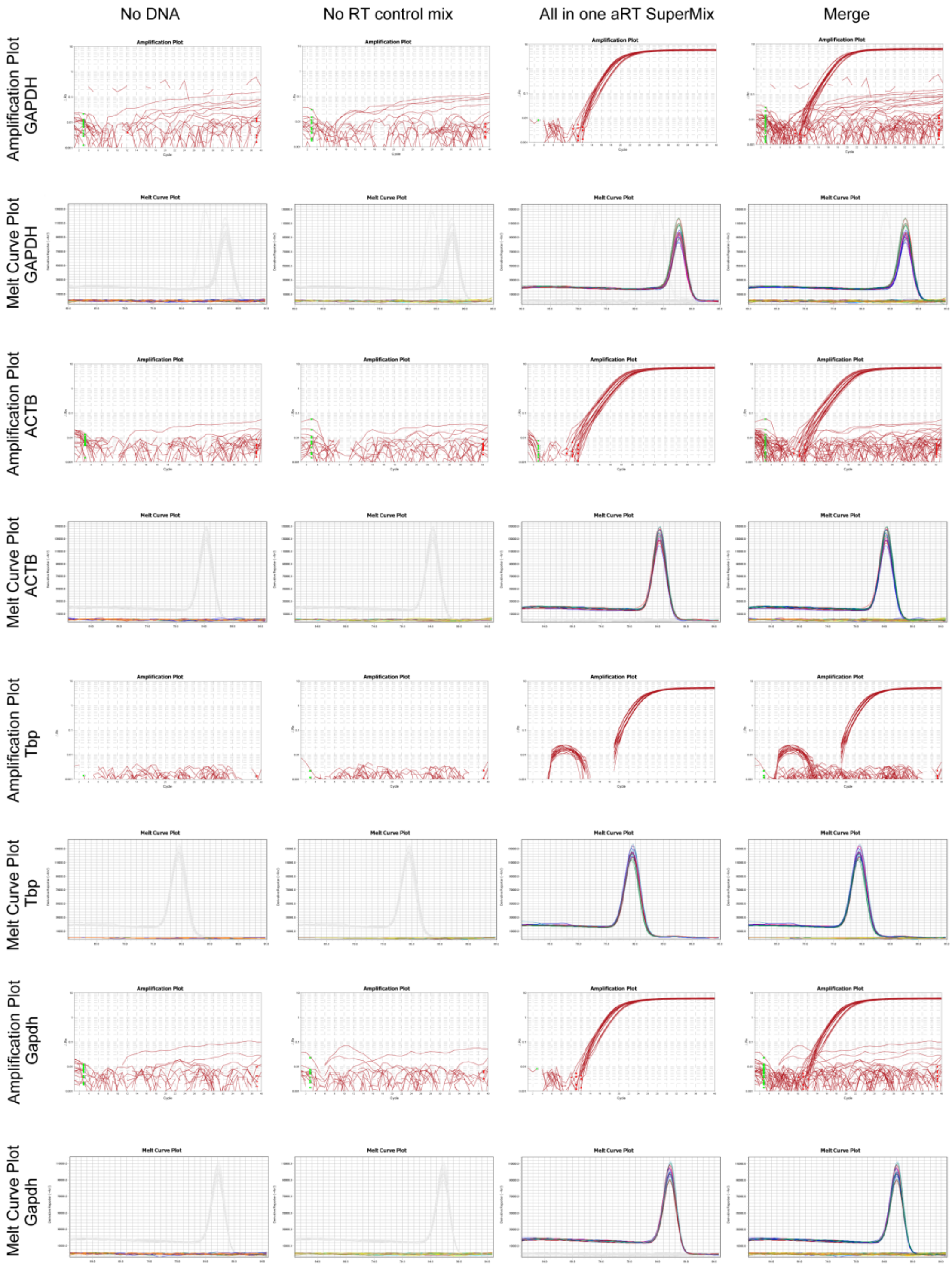

Supplementary Data Figure 6: Negative controls of qRT-PCR.

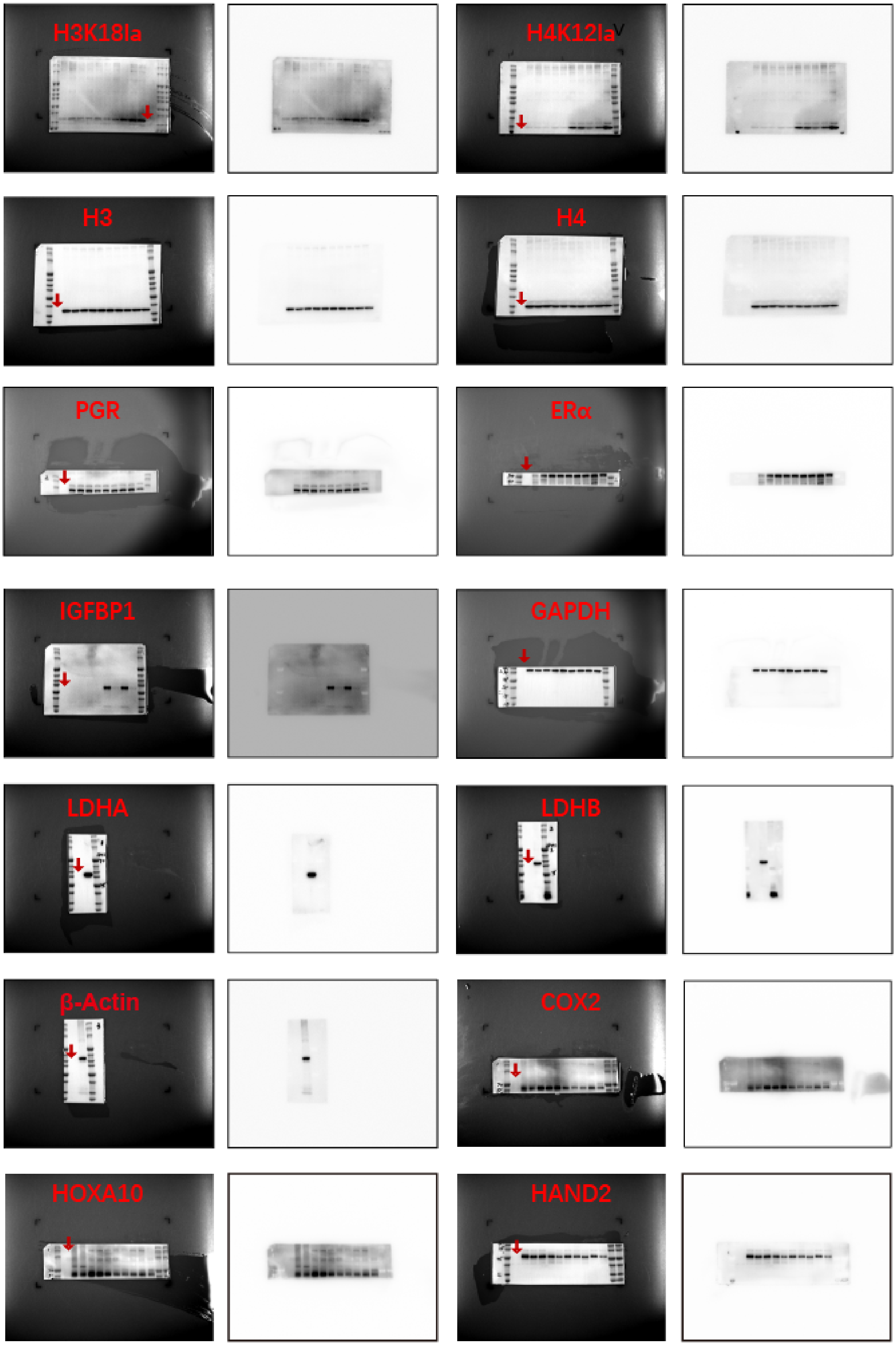

Supplementary Data Figure 7: Negative controls of WB. Representative Western blot image showing the target protein in experimental samples and its absence in the

negative control (indicated by the red arrow).

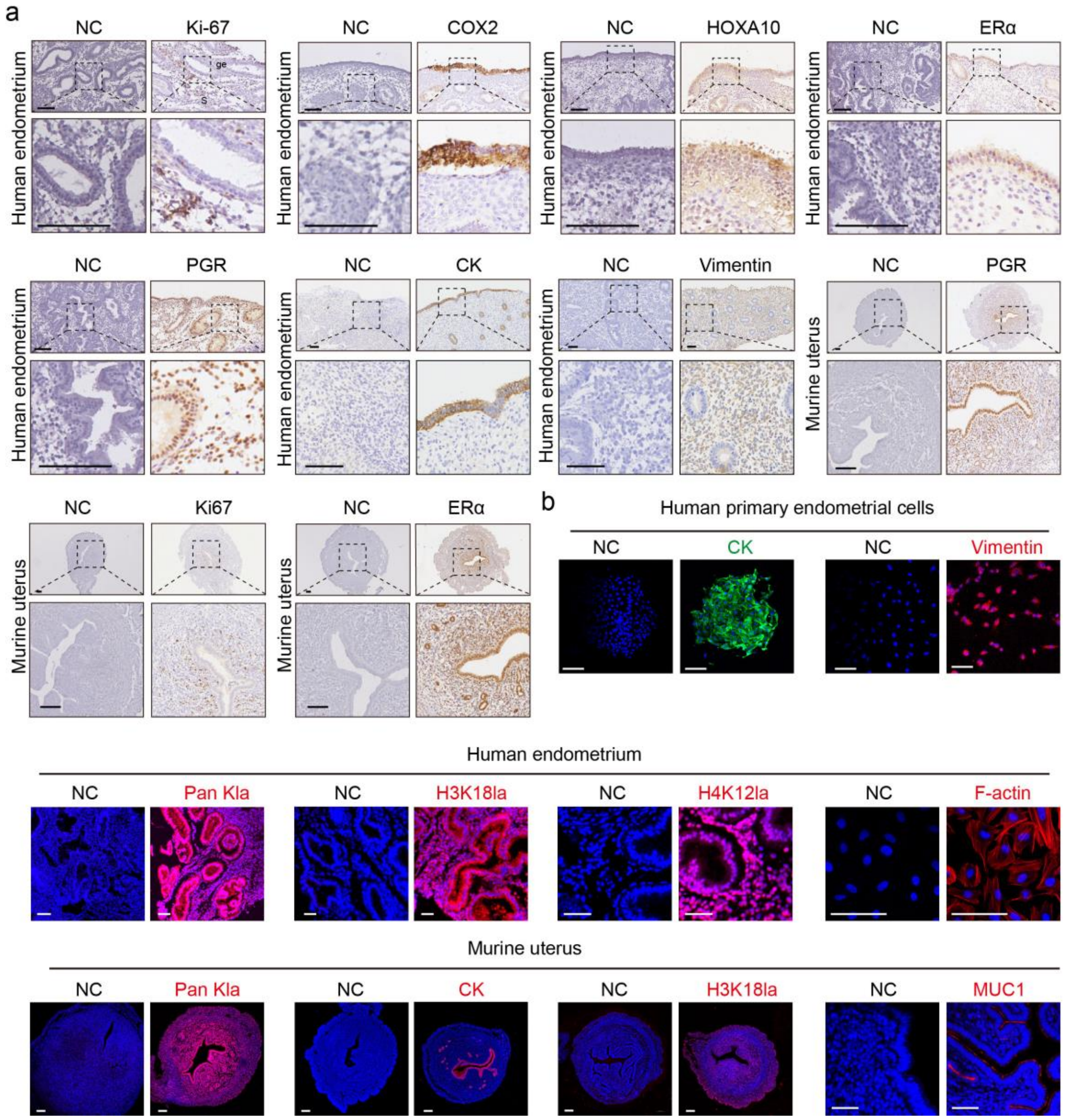

Supplementary Data Figure 8: Negative controls of IHC and IF staining. a, Representative images of IHC staining along with negative controls. **b**, Representative images of IF staining along with negative controls. Images are representative of four independent biological replicates. Scale bar: 100 μm.

a

Ages and BMIs of the two groups before and after matching
(donors of endometrial samples used for qRT-PCR)

Item	Before PSM Control group (n=25)	Before PSM PCOS group (n=12)	Before PSM *P* value	After PSM Control group (n=11)	After PSM PCOS group (n=11)	After PSM *P* value
Age of female (year)	32.5±3.1	31.6±3.7	0.396	32.5±3.1	32.1±3.2	0.752
BMI (kg/m²)	21.1±3.0	24.4±3.3	0.004	23.9±2.3	24.9±2.1	0.083

b

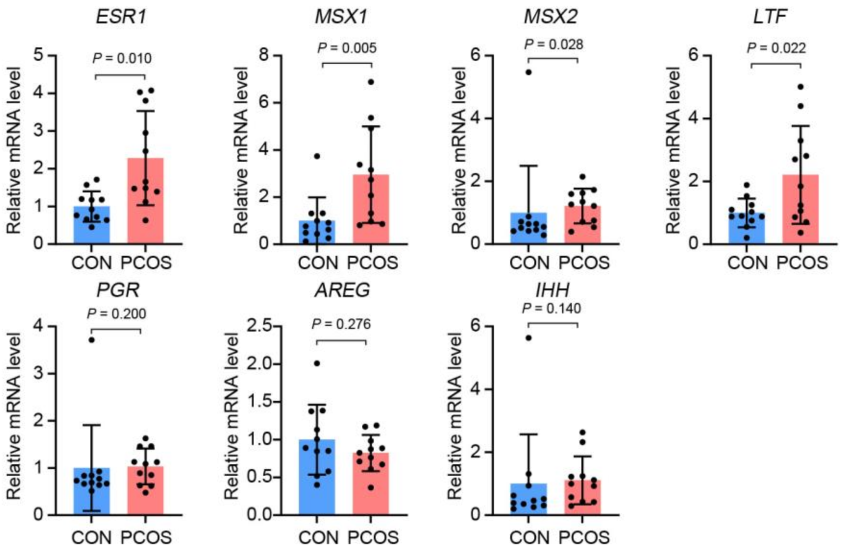

c

Ages and BMIs of the two groups before and after matching
(donors of endometrial samples used for WB)

Item	Before PSM Control group (n=25)	Before PSM PCOS group (n=12)	Before PSM *P* value	After PSM Control group (n=6)	After PSM PCOS group (n=6)	After PSM *P* value
Age of female (year)	32.5±3.1	31.6±3.7	0.396	32.2±2.8	32.0±3.5	0.912
BMI (kg/m²)	21.1±3.0	24.4±3.3	0.004	23.8±2.5	24.1±2.3	0.831

d

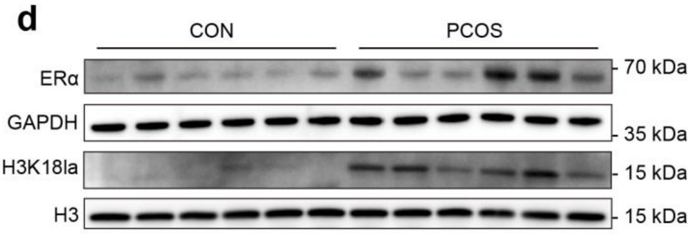

e

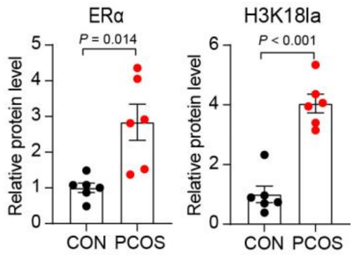

Supplementary Data Figure 9: Elevated endometrial ERα and H3K18la levels are PCOS-dependent but not BMI-dependent. a, Ages and BMIs of the two groups before and after matching (donors of endometrial samples used for qRT-PCR). **b**, Relative mRNA levels of *ESR1*, *MSX1*, *MSX2*, *LTF*, *PGR*, *AREG*, and *IHH* in the

human mid-secretory endometrium after PSM (*n* = 11, biologically independent samples). **c**, Ages and BMIs of the two groups before and after matching (donors of endometrial samples used for WB). **d**-**e**, Protein levels of ERα and H3K18la in the human mid-secretory endometrium after PSM (*n* = 6, biologically independent samples). PSM was performed using BMI as the matching variable.

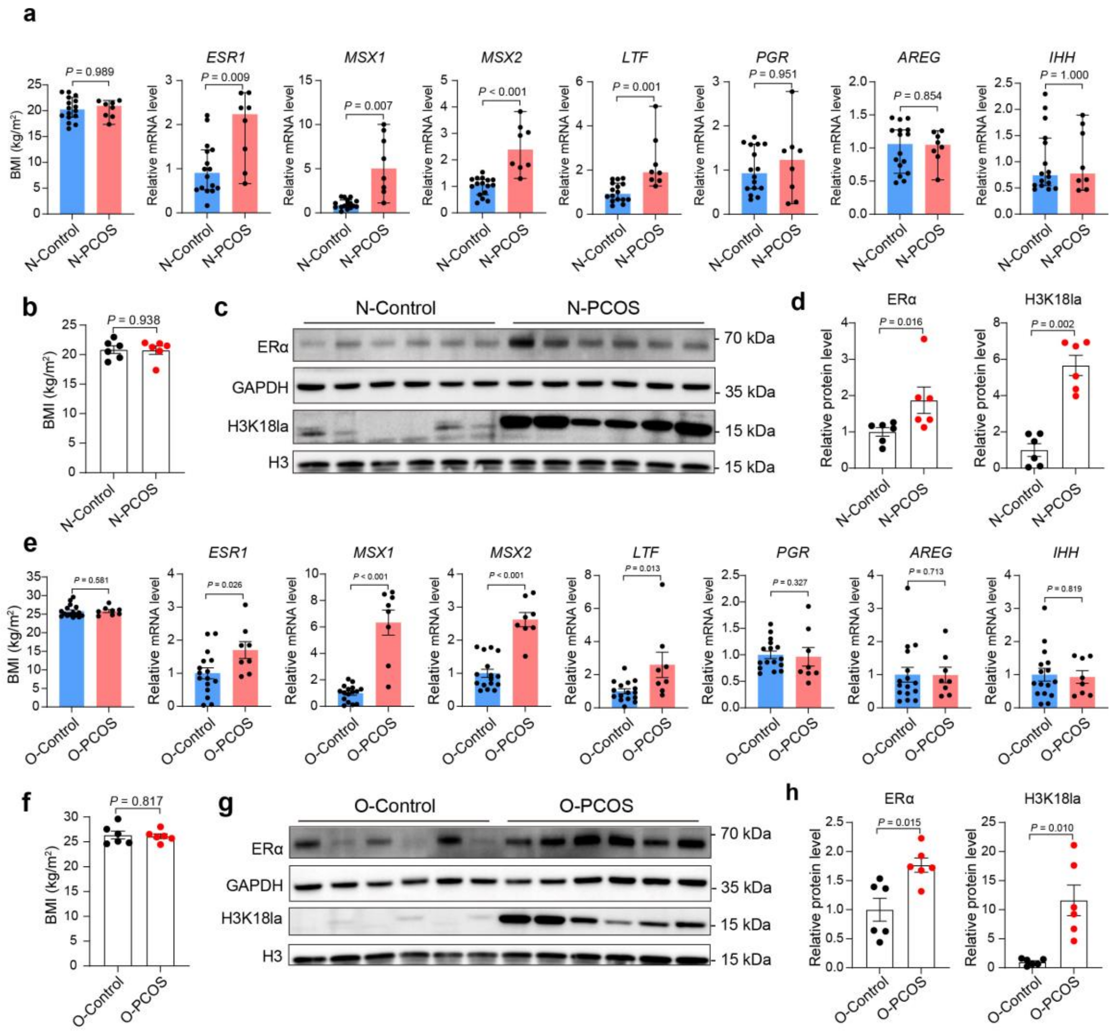

Supplementary Data Figure 10: Endometrial ERα and H3K18la levels are consistently upregulated in PCOS compared to controls with matched BMIs. a, BMIs of donors and relative mRNA levels of *ESR1*, *MSX1*, *MSX2*, *LTF*, *PGR*, *AREG*, and *IHH* in the human mid-secretory endometrium of normal-weight controls (N-Control) and PCOS (N-PCOS) (n = 16 for N-Control and n = 8 for N-PCOS, biologically independent samples). **b-d**, BMIs of donors and protein levels of ERα and H3K18la in the human mid-secretory endometrium of N-Control and N-PCOS (n = 6, biologically independent samples). **e**, BMIs of donors and relative mRNA levels of *ESR1*, *MSX1*, *MSX2*, *LTF*, *PGR*, *AREG*, and *IHH* in the human mid-secretory endometrium of obese controls (O-Control) and PCOS (O-PCOS) (n = 16 for O-Control and n = 8 for O-PCOS, biologically independent samples). **f-h**, BMIs of donors and protein levels of ERα and H3K18la in the human mid-secretory endometrium of O-Control and O-PCOS (n = 6, biologically independent samples).
