# Supplementary material for: Women with polycystic ovary syndrome exhibit impaired endometrial receptivity with excessive ERα and histone lactylation
Source: Nat Commun. 2026 Jan 21;17:1739. doi: 10.1038/s41467-026-68441-0 (PMC12913789; doi:10.1038/s41467-026-68441-0)
Supplement: Supplementary file 4 — Reporting Summary [file 41467_2026_68441_MOESM4_ESM.pdf]

nature portfolio

Corresponding author(s): Heng Pan, Rong Li, Yang Yu, Ping Zhou

Last updated by author(s): Nov 12, 2025

Reporting Summary

Nature Portfolio wishes to improve the reproducibility of the work that we publish. This form provides structure for consistency and transparency in reporting. For further information on Nature Portfolio policies, see our Editorial Policies and the Editorial Policy Checklist.

Statistics

For all statistical analyses, confirm that the following items are present in the figure legend, table legend, main text, or Methods section.

n/a	Confirmed	
☐	☒	The exact sample size (*n*) for each experimental group/condition, given as a discrete number and unit of measurement
☐	☒	A statement on whether measurements were taken from distinct samples or whether the same sample was measured repeatedly
☐	☒	The statistical test(s) used AND whether they are one- or two-sided<br>*Only common tests should be described solely by name; describe more complex techniques in the Methods section.*
☐	☒	A description of all covariates tested
☐	☒	A description of any assumptions or corrections, such as tests of normality and adjustment for multiple comparisons
☐	☒	A full description of the statistical parameters including central tendency (e.g. means) or other basic estimates (e.g. regression coefficient) AND variation (e.g. standard deviation) or associated estimates of uncertainty (e.g. confidence intervals)
☐	☒	For null hypothesis testing, the test statistic (e.g. *F*, *t*, *r*) with confidence intervals, effect sizes, degrees of freedom and *P* value noted<br>*Give P values as exact values whenever suitable.*
☒	☐	For Bayesian analysis, information on the choice of priors and Markov chain Monte Carlo settings
☒	☐	For hierarchical and complex designs, identification of the appropriate level for tests and full reporting of outcomes
☒	☐	Estimates of effect sizes (e.g. Cohen's *d*, Pearson's *r*), indicating how they were calculated

Our web collection on statistics for biologists contains articles on many of the points above.

Software and code

Policy information about availability of computer code

Data collection	Immunofluorescence results were obtained by ZEISS LSM880-LASER SCANNING CONFOCAL. The H&E and immunohistochemistry results were collected by NIS-Elements 3.2. CUT&Tag data were collected by the Illumina NovaSeq 6000 platform. Flow cytometry data were acquired on the BD LSRFortessa™ X-20 Cell Analyzer. Cell viability were carried out with the Sartorius Incucyte S3.
Data analysis	fastp (version 0.20.1), HISAT2 (version 2.1.0), HTSeq-count (version 0.11.2), DESeq2 (version 1.22.2), python (version 3.10.8), Bowtie2 (version 2.5.1), MACS2 (version 2.2.9.1), ChIPseeker (version 1.36.0), Homer (version 4.11), R (version 4.3.1), Diffbind (version 3.12.0), GraphPad Prism (version 9.5.1), GSEA (version 4.3.2), SPSS(26.0), ImageJ (version 1.53v).

For manuscripts utilizing custom algorithms or software that are central to the research but not yet described in published literature, software must be made available to editors and reviewers. We strongly encourage code deposition in a community repository (e.g. GitHub). See the Nature Portfolio guidelines for submitting code & software for further information.

Data

Policy information about availability of data

All manuscripts must include a data availability statement. This statement should provide the following information, where applicable:

- Accession codes, unique identifiers, or web links for publicly available datasets
- A description of any restrictions on data availability
- For clinical datasets or third party data, please ensure that the statement adheres to our policy

Source data are provided with this paper. The sequencing data generated in this study have been deposited in the Genome Sequence Archive (accession number HRA009852). BED files of CUT&Tag data have been uploaded to the Open Archive for Miscellaneous Data (accession number OMIX012938). The human reference genome hg38 can be accessed in the National Center for Biotechnology Information (NCBI) (https://ftp.ncbi.nlm.nih.gov/genomes/all/GCF/000/001/405/GCF_000001405.39_GRCh38.p13/).

Research involving human participants, their data, or biological material

Policy information about studies with human participants or human data. See also policy information about sex, gender (identity/presentation), and sexual orientation and race, ethnicity and racism.

Reporting on sex and gender	Endometrial samples of both control and PCOS women.
Reporting on race, ethnicity, or other socially relevant groupings	No socially relevant groupings were included.
Population characteristics	Endometrial tissues were collected from women aged ≤ 40 years during proliferative, mid-secretory, or other menstrual phases, with cycle staging confirmed by serum LH peak detection and transvaginal ultrasound-verified ovulation, followed by formalin fixation, paraffin embedding, and H&E staining, and final histological dating was independently validated by two senior pathologists.
Recruitment	All endometrial samples were obtained with the informed consent of the willing patients, with the control group comprising women undergoing IVF treatment due to male or tubal factors, who exhibited regular menstrual cycles (21–35 days). Polycystic ovary syndrome (PCOS) was diagnosed based on the 2003 Rotterdam criteria. Exclusion criteria included: Presence of endometriosis, RIF or RSA, 2) Thyroid disease, 3) Severe intrauterine adhesion, 4) Severe uterine abnormality.
Ethics oversight	This study was approved by the Ethics Committee of Reproductive Medicine, Peking University Third Hospital (No. 2023-192-02).

Note that full information on the approval of the study protocol must also be provided in the manuscript.

Field-specific reporting

Please select the one below that is the best fit for your research. If you are not sure, read the appropriate sections before making your selection.

☒ Life sciences ☐ Behavioural & social sciences ☐ Ecological, evolutionary & environmental sciences

For a reference copy of the document with all sections, see nature.com/documents/nr-reporting-summary-flat.pdf

Life sciences study design

All studies must disclose on these points even when the disclosure is negative.

Sample size	No statistical methods were used to pre-determine sample sizes, but our sample sizes were similar to those reported in previous publications (PMID: 38795132, PMID: 37270588, PMID: 36693099).
Data exclusions	No data were excluded in this study.
Replication	Each experiment was repeated at least four times and all attempts at replication were successful.
Randomization	We acknowledge the inherent differences in baseline characteristics (e.g., BMI, FSH,LH and A levels) between PCOS women and controls due to the disease pathology. Therefore, we selected age-matched controls and applied appropriate statistical adjustments for confounding factors in subsequent analyses. Murine samples were randomly allocated to experimental groups.
Blinding	Blinding design was not necessary in this study since the sequencing and evaluation processes were equally for all the human endometrial samples and murine uterine samples.

Reporting for specific materials, systems and methods

We require information from authors about some types of materials, experimental systems and methods used in many studies. Here, indicate whether each material, system or method listed is relevant to your study. If you are not sure if a list item applies to your research, read the appropriate section before selecting a response.

Materials & experimental systems

n/a	Involved in the study
☐	☒ Antibodies
☐	☒ Eukaryotic cell lines
☒	☐ Palaeontology and archaeology
☐	☒ Animals and other organisms
☐	☒ Clinical data
☒	☐ Dual use research of concern
☒	☐ Plants

Methods

n/a	Involved in the study
☐	☒ ChIP-seq
☐	☒ Flow cytometry
☒	☐ MRI-based neuroimaging

Antibodies

Antibodies used

IGFBP1 (D4E9T) XP® Rabbit mAb, Cell signaling Technology, USA, Cat#31025T , 1:1000 dilution.
Progesterone Receptor A/B (D8Q2J) XP® Rabbit mAb, Cell signaling Technology, USA, Cat#8757S, 1:1000 dilution.
Anti-Estrogen Receptor alpha antibody [E115] - ChIP Grade, Abcam, UK, Cat#ab32063, 1:1000 dilution.
Anti-COX2 / Cyclooxygenase 2 antibody [EPR12012], Abcam, UK, Cat#ab179800, 1:200 dilution.
Anti-HOXA10 antibody, Abcam, UK, Cat#ab191470, 1:100 dilution.
Anti-Ki67 antibody [SP6], Abcam, UK, Cat#ab16667, 1:200 dilution.
Anti-MUC1 antibody [EPR1023], Abcam, UK, Cat#ab109185, 1:200 dilution.
Anti-Cytokeratin 19 antibody [EP1580Y] - Cytoskeleton Marker, Abcam, UK,Cat#ab52625, 1:10000 dilution.
Anti-Vimentin antibody [RV202] - Cytoskeleton Marker, Abcam, UK, Cat#ab8978, 1:1000 dilution.
Anti-L-Lactyl Lysine Rabbit pAb, PTM Biolabs, China, Cat#PTM-1401, IF: 1:50 dilution, WB: 1:1000 dilution.
Anti-L-Lactyl-Histone H3 (Lys18) Rabbit mAb-ChIP Grade, PTM Biolabs, China, Cat#PTM-1427RM, Cut&tag:1:50 dilution.
Anti-L-Lactyl-Histone H3 (Lys18) Rabbit mAb, PTM Biolabs, China, Cat#PTM1406RM, 1:1000 dilution.
Anti-H4K12la Rabbit pAb,PTM Biolabs, China, Cat#PTM1411RM, 1:1000 dilution.
Anti-Histone H4 Rabbit pAb (CT), PTM Biolabs, China, Cat#PTM1015RM, 1:2000 dilution.
Anti-Histone H3 Rabbit mAb (CT), PTM Biolabs, China, Cat#PTM1002RM, 1:2500 dilution.
ERα Mouse mAb, Immunoway, USA, Cat#YM0252, 1:200 dilution.
LDHB Polyclonal antibody, Proteintech, USA, Cat#14824-1-AP, 1:10000 dilution.
LDHA-Specific Polyclonal antibody, Proteintech, USA, Cat#19987-1-AP, 1:5000 dilution.
Anti-GAPDH antibody [EPR16891] – Loading Control, Abcam, UK, Cat#ab181602, 1:10000 dilution.
Anti-beta Actin antibody [AC-15], Abcam, UK, Cat#ab6276, 1:10000 dilution.
HRP-labeled Donkey Anti-Goat IgG(H+L), Beyotime, China, Cat#A0181, 1:1000 dilution.
HRP-labeled Goat Anti-Rabbit IgG(H+L), Beyotime, China, Cat#A0208, 1:1000 dilution.
HRP-labeled Goat Anti-Mouse IgG(H+L), Beyotime, China, Cat#A0216, 1:1000 dilution.
Phalloidin-iFluor 555 reagent, Abcam, UK, Cat#ab176756, 1:200 dilution.
Phalloidin-iFluor 488 reagent, Abcam, UK, Cat#ab176753,1:200 dilution.

Validation

Rabbit anti-IGFBP1 antibody https://www.cellsignal.cn/products/primary-antibodies/igfbp1-d4e9t-xp-rabbit-mab/31025
Rabbit anti-Progesterone Receptor antibody https://www.cellsignal.cn/products/primary-antibodies/progesterone-receptor-a-b-d8q2j-xp-rabbit-mab/8757
Rabbit anti-Estrogen Receptor alpha antibody https://www.abcam.cn/products/primary-antibodies/estrogen-receptor-alpha-antibody-e115-chip-grade-ab32063.html
Rabbit anti-COX2 antibody https://www.abcam.cn/products/primary-antibodies/cox2--cyclooxygenase-2-antibody-epr12012-ab179800.html
Goat anti-HOXA10 antibody https://www.abcam.cn/products/primary-antibodies/hoxa10-antibody-ab191470.html
Rabbit anti-Ki67 antibody https://www.abcam.cn/products/primary-antibodies/ki67-antibody-sp6-ab16667.html
Rabbit anti-MUC1antibody https://www.abcam.com/en-us/products/primary-antibodies/muc1-antibody-epr1023-ab109185
Rabbit anti-Cytokeratin antibody https://www.abcam.cn/products/primary-antibodies/cytokeratin-19-antibody-ep1580y-cytoskeleton-marker-ab52625.html
Mouse anti-Vimentin antibody https://www.abcam.com/en-us/products/primary-antibodies/vimentin-antibody-rv202-cytoskeleton-marker-ab8978#
Rabbit anti-pan Kla antibody http://www.ptm-biolab.com.cn/productDetail.html?id=5862
Rabbit anti-H3K18la antibody ChIP Grade http://www.ptm-biolab.com.cn/productDetail.html?id=8046
Rabbit anti-H3K18la antibody http://www.ptm-biolab.com.cn/productDetail.html?id=5558
Rabbit anti-H4K12la antibody http://www.ptm-biolab.com.cn/productDetail.html?id=5193
Rabbit anti-Histone H4 antibody http://www.ptm-biolab.com.cn/productDetail.html?id=4603
Rabbit anti-Histone H3 antibody http://www.ptm-biolab.com.cn/productDetail.html?id=6697
Mouse anti-ERα Antibody https://www.immunoway.com.cn/products/primary-antibodies/YM0252-ERa-Mouse-mAb.html
Rabbit anti-LDHB antibody https://www.ptgcn.com/products/LDHB-Antibody-14824-1-AP.htm
Rabbit anti-LDHA antibody https://www.ptgcn.com/products/LDHA-Specific-Antibody-19987-1-AP.htm
Rabbit anti-GAPDH antibody https://www.abcam.cn/products/primary-antibodies/gapdh-antibody-epr16891-loading-control-

ab181602.html
Mouse anti-beta Actin antibody https://www.abcam.cn/products/primary-antibodies/beta-actin-antibody-ac-15-ab6276.html
HRP-conjugated Affinipure Donkey anti-Goat IgG(H+L) https://www.beyotime.com/product/A0181.htm
HRP-conjugated Affinipure Goat Anti-Rabbit IgG(H+L) https://www.beyotime.com/product/A0208.htm
HRP-conjugated Affinipure Goat Anti-Mouse IgG(H+L) https://www.beyotime.com/product/A0216.htm
Phalloidin-iFluor 555 Reagent https://www.abcam.cn/products/assay-kits/phalloidin-ifluor-555-reagent-ab176756.html
Phalloidin-iFluor 488 Reagent https://www.abcam.cn/products/assay-kits/phalloidin-ifluor-488-reagent-ab176753.html

Eukaryotic cell lines

Policy information about cell lines and Sex and Gender in Research

Cell line source(s)	The ishikawa cells (CL-0283) were purchased from Wuhan Pricella cell Life Science & Technology Co., Ltd.
Authentication	Ishikawa cells were regularly authenticated by morphological observation and short tandem repeat (STR) analysis.
Mycoplasma contamination	Ishikawa cells were tested for the absence of mycoplasma contamination.
Commonly misidentified lines (See ICLAC register)	No commonly misidentified lines exist.

Animals and other research organisms

Policy information about studies involving animals; ARRIVE guidelines recommended for reporting animal research, and Sex and Gender in Research

Laboratory animals	C57BL/6J female mice at 3 weeks of age were obtained from Beijing Vital River Laboratory Animal Technology (Beijing, China). All mice always had access to food, water and were raised in a controlled environment with a 12-hour light/dark cycle, at a room temperature of 20 - 25°C and with a humidity of 55% ± 10%. 3-week-old C57BL/6J female mice were given a daily subcutaneous injection of DHEA (6 mg/100 g body weight; D4000-10 g, Sigma Aldrich, USA) dissolved in 0.1 mL of sesame oil for 21 days to establish the PCOS mouse model.
Wild animals	No wild animals were used in this study.
Reporting on sex	All the mice were female.
Field-collected samples	No field-collected samples were used in this study.
Ethics oversight	All of the experimental procedures followed the guidelines of NIH for the Care and Use of Laboratory Animals. This study was approved by the Ethics Committee of Reproductive Medicine, Peking University Third Hospital (approved number:A2022111)

Note that full information on the approval of the study protocol must also be provided in the manuscript.

Clinical data

Policy information about clinical studies
All manuscripts should comply with the ICMJE guidelines for publication of clinical research and a completed CONSORT checklist must be included with all submissions.

Clinical trial registration	As a retrospective study, no clinical trial registration was performed.
Study protocol	The clinical data used in this study was from a retrospective observational study design. We collected the clinical information of 4,278 patients who had undergone IVF/ICSI treatments at the Center for Reproductive Medicine of Peking University Third Hospital. After strictly controlling for embryo factor and adjusting for potential confounders using propensity score matching (PSM), the rates of positive pregnancy rate, implantation rate, and live birth rate were significantly lower in PCOS women.
Data collection	The baseline characteristics and pregnancy outcomes were collected from both the control group and PCOS patients undergoing IVF/ICSI at the Reproductive Medical Center of Peking University Third Hospital.
Outcomes	The primary outcome was implantation rate. The secondary outcomes were positive pregnancy rate, and live birth rate.

Plants

Seed stocks	n/a
Novel plant genotypes	n/a
Authentication	n/a

ChIP-seq

Data deposition

☒ Confirm that both raw and final processed data have been deposited in a public database such as GEO.

☒ Confirm that you have deposited or provided access to graph files (e.g. BED files) for the called peaks.

Data access links<br>*May remain private before publication.*	CUT&Tag data have been uploaded to the Genome Sequence Archive (accession number: HRA009852).
Files in database submission	Endometrium-H3K18la-CON-1.R1.fq.gz<br>Endometrium-H3K18la-CON-1.R2.fq.gz<br>Endometrium-H3K18la-CON-2.R1.fq.gz<br>Endometrium-H3K18la-CON-2.R2.fq.gz<br>Endometrium-H3K18la-CON-3.R1.fq.gz<br>Endometrium-H3K18la-CON-3.R2.fq.gz<br>Endometrium-H3K18la-CON-4.R1.fq.gz<br>Endometrium-H3K18la-CON-4.R2.fq.gz<br>Endometrium-H3K18la-PCOS-1.R1.fq.gz<br>Endometrium-H3K18la-PCOS-1.R2.fq.gz<br>Endometrium-H3K18la-PCOS-2.R1.fq.gz<br>Endometrium-H3K18la-PCOS-2.R2.fq.gz<br>Endometrium-H3K18la-PCOS-3.R1.fq.gz<br>Endometrium-H3K18la-PCOS-3.R2.fq.gz<br>Endometrium-H3K18la-PCOS-4.R1.fq.gz<br>Endometrium-H3K18la-PCOS-4.R2.fq.gz<br>CON and PCOS endometrial bed file.zip
Genome browser session<br>(e.g. UCSC)	No genome browser session was available.

Methodology

Replicates	Each experiment was repeated at least four times.
Sequencing depth	Purified PCR products were assessed using the Agilent 2100 Bioanalyzer. Libraries were sequenced on the Illumina NovaSeq 6000 platform, generating 150 bp paired-end reads.
Antibodies	Anti-L-Lactyl-Histone H3 (Lys18) Rabbit mAb-ChIP Grade, PTM Biolabs, China, Cat#PTM-1427RM, Cut&tag:1:50 dilution.
Peak calling parameters	Peak calling was performed by MACS2 with the “narrowPeak” parameter. Normalization was performed with the RPGC method.
Data quality	The raw data was trimmed by fatsp. Trimmed reads were aligned to the human reference genome hg38 using Bowtie2.
Software	fastp (version 0.20.1), Bowtie2 (version 2.5.1), MACS2 (version 2.2.9.1), ChIPseeker (version 1.36.0), Homer (version 4.11), Diffbind (version 3.12.0).

Flow Cytometry

Plots

Confirm that:

☒ The axis labels state the marker and fluorochrome used (e.g. CD4-FITC).

☒ The axis scales are clearly visible. Include numbers along axes only for bottom left plot of group (a 'group' is an analysis of identical markers).

☒ All plots are contour plots with outliers or pseudocolor plots.

☒ A numerical value for number of cells or percentage (with statistics) is provided.

Methodology

Sample preparation	Endometrial samples were placed into centrifuge tubes filled with sterile Dulbecco phosphate-buffered saline (DPBS) and immediately transported to a clean bench on ice. After washing with DPBS supplemented with 200 U/mL penicillin and 200 μg/mL streptomycin until the blood was cleaned as much as possible, the samples were cut into 1 mm3 small blocks. The fragments were transferred to tubes with 2 mg/ml collagenase type I and incubated for 1 h at 37◦C in 5% CO2, followed by treatment with DNase I for 30 min with shaking every 10 min during digestion. The obtained cellular suspension was sequentially passed through 100-μm and 40-μm cell strainers stacked together with a dish underneath. Because of the different cellular sizes, endometrial epithelial cells (EECs) remained on the 40-μm strainer, whereas endometrial stromal cells (ESCs) were passed through the two strainers and reached the dish. The EECs were then resuspended in DMEM/F12 medium (without phenol red) supplemented with 10% fetal bovine serumand plated in 35 mm dishes . The ESCs were resuspended after the red blood cells were lysed using ACK lysis buffer and incubated. were purchased from Wuhan Procell Life Science & Technology Co., Ltd.
Instrument	BD LSRFortessa™ X-20 Cell Analyzer
Software	FlowJo_V10
Cell population abundance	The primary endometrial cells isolated by cell strainer filtration were subjected to 7-AAD staining for viable/dead cell discrimination.
Gating strategy	The gating strategy was performed as follows: First, the intact cell population was identified based on forward scatter (FSC) and side scatter (SSC) characteristics, where the preliminary gate was set to exclude debris and cell aggregates. Subsequently, viable cells were discriminated from dead cells by establishing the boundary between 7-AAD-negative (viable) and 7-AAD-positive (non-viable) populations, with the threshold determined using unstained controls. This two-step gating approach ensured analysis of only viable, single cells in all experiments.

☒ Tick this box to confirm that a figure exemplifying the gating strategy is provided in the Supplementary Information.
